# Gonorrhoea: a systematic review of prevalence reporting globally

**DOI:** 10.1186/s12879-021-06381-4

**Published:** 2021-11-11

**Authors:** Jane Whelan, Victoria Abbing-Karahagopian, Laura Serino, Magnus Unemo

**Affiliations:** 1 Clinical and Epidemiology Research and Development, GSK, Amsterdam, The Netherlands; 2 Clinical and Epidemiology Research and Development, GSK, Siena, Italy; 3 WHO Collaborating Centre for Gonorrhoea and other STIs, Department of Laboratory Medicine, Microbiology, Faculty of Medicine and Health, Örebro University, Örebro, Sweden

**Keywords:** global, gonorrhoea, *Neisseria gonorrhoeae*, prevalence, systematic review, men-who-have-sex-with-men, sex workers

## Abstract

**Background:**

The World Health Organization (WHO) recommends periodic gonorrhoea prevalence assessments in the general population or proxies thereof (including pregnant women, women attending family planning clinics, military recruits, and men undergoing employment physicals for example) and in population groups at increased risk, including men-who-have-sex-with-men (MSM) and sex workers.

**Method:**

We evaluated reported prevalence data, including estimates from proxy general population samples to reflect the WHO recommendations. We describe the outcomes from the general population country-by-country and extend previous reviews to include MSM, sex workers, and extragenital infections.

**Result and conclusion:**

In our systematic search, 2015 titles were reviewed (January 2010–April 2019) and 174 full-text publications were included. National, population-based prevalence data were identified in only four countries (the United States of America, the United Kingdom, Peru, New Caledonia) and local population-based estimates were reported in areas within five countries (China, South Africa, Brazil, Benin, and Malawi). The remaining studies identified only reported test positivity from non-probability, proxy general population samples. Due to the diversity of the reviewed studies, detailed comparison across studies was not possible. In MSM, data were identified from 64 studies in 25 countries. Rectal infection rates were generally higher than urogenital or pharyngeal infection rates, where extragenital testing was conducted. Data on sex workers were identified from 41 studies in 23 countries; rates in female sex workers were high. Current prevalence monitoring was shown to be highly suboptimal worldwide. Serial prevalence monitoring of critical epidemiological variables, and guidelines to optimize prevalence study conduct and reporting beyond antenatal settings are recommended.

**Supplementary Information:**

The online version contains supplementary material available at 10.1186/s12879-021-06381-4.

## Background

Gonorrhoea is a sexually transmitted infection (STI) caused by *Neisseria gonorrhoeae* (the gonococcus). In 2016, an estimated 87 million incident cases occurred among persons aged 15–49 years worldwide with an incidence rate of 20 cases/1000 women and 26/1000 men [1].

Gonorrhoea affects the urogenital tract, oropharynx, rectum, or conjunctiva, and repeat infections are common. Urogenital infections are often asymptomatic, particularly in women, but irrespective of symptoms, gonorrhoea is associated with substantial morbidity. Serious complications and sequelae include pelvic inflammatory disease, chronic pelvic pain, ectopic pregnancy, and infertility in women [2]. Infection during pregnancy is also associated with low birth weight and neonatal conjunctivitis, which can progress to blindness [2, 3]. In men, gonorrhoea can cause epididymitis [2]. Rectal and pharyngeal gonorrhoea cases, mostly asymptomatic, are prevalent in men-who-have-sex-with-men (MSM), but can be common also in women and, particularly pharyngeal infection, in men who have sex only with women [4]. The presence of gonorrhoea is also a co-factor in human immunodeficiency virus (HIV) transmission [5].

Gonorrhoea is substantially underdiagnosed and underreported worldwide [3]. Even in high-income economies with well-established STI surveillance systems, it is estimated that more than half of infections are unidentified or unreported [6, 7]. This underdiagnosis/underreporting is higher in less-resourced settings and settings using syndromic management with limited access to state-of-the-art diagnostics such as nucleic acid amplification tests (NAATs). Though partially explained by the asymptomatic nature of the infection, underreporting is also due to delays in seeking healthcare and inaccessible or inadequate STI testing/treatment in underserved populations or those particularly vulnerable to infection: adolescents and young people, some ethnic and racial groups, communities of lower socioeconomic status, MSM, sex workers, and others [8].

The World Health Organization (WHO)’s global target is a 90% reduction in gonorrhoea cases by 2030 [9]. To monitor progress towards this goal, STI trend monitoring at the national level is recommended. This should include routine prevalence assessments (every two to three years) of bacterial STIs among general populations of men and women (e.g. including pregnant women, women attending family planning clinics, military recruits and men undergoing employment physicals) [3]. Monitoring in high-risk priority populations including MSM and sex workers is also recommended [3, 9].

The WHO reports prevalence estimates of curable non-viral STIs at a global and regional level using epidemic models, while recognizing the small number of prevalence data points that are available to generate reliable estimate [1, 3]. Notably, for key population groups such as MSM and sex workers, who likely contribute substantially to the worldwide infection burden, gonorrhoea prevalence in global estimates is indirectly accounted for [1] and estimates do not reflect rectal and pharyngeal infection.

*N. gonorrhoeae* is progressively developing antimicrobial resistance (AMR) to all therapeutic antibiotics, and the WHO has issued warnings that untreatable gonorrhoea may be on the horizon [10]. National prevalence estimates are an essential indicator of the state of gonorrhoea and STI control at state level and globally [3]. In this review, we aimed to evaluate global prevalence reporting in the general population, and proxies thereof, on a country-by-country basis, extending previous reviews to report on key population groups of MSM and female and male sex workers (FSW and MSW), including extragenital as well as urogenital infection.

## Methods

### Search strategy and selection criteria

We conducted a systematic search of PubMed following PRISMA guidelines (Additional file 1) for papers published from 1 January 2010 to 11 April 2019. We derived a sensitive search strategy requiring at least one medical subject headings (MeSH) term related to a sexually transmitted disease (STD) or gonorrhoea and at least one reference to the keyword ‘gonorrhoea’ in the title or abstract. We did not specify the population (e.g. MSM, FSW or MSW), as we noted substantial overlap in reporting of risk groups and inclusion of terms such as ‘prevalence’, ‘epidemiology’ or ‘rate’ rendered the search too specific, omitting relevant papers (Additional file 2). Two authors (JW and VAK) independently screened all titles and abstracts against pre-specified inclusion and exclusion criteria (Additional file 3) and agreed on the selection of articles to be obtained as full text. English-language abstracts were reviewed but the full text was translated as necessary, from Portuguese, Spanish, and Chinese, where relevant. The systematic search was supplemented with an online English-language country-by-country search of websites, data repositories and surveillance reports of public health and/or governmental agencies using the country name, and ‘gonorr*’ or ‘sexually transmitted’ and ‘disease’ or ‘infection’ to identify data sources and provide context to prevalence estimates. We reviewed regional and international health agency data (WHO, European Centre for Disease Prevention and Control [ECDC]) and contacted relevant experts in the field. AMR monitoring, an essential component of gonorrhoea surveillance [10] and worthy of a separate review, was beyond the scope of this search.

### Data analysis

The primary outcome (prevalence of gonorrhoea) was defined as the proportion of persons with laboratory-confirmed (culture and/or NAAT positive) gonorrhoea in the population within a specified time. It became apparent early in the literature search that population-based prevalence estimates were very limited and so to address the WHO recommendation to derive estimates from studies which are not necessarily population-based but nevertheless relevant, we defined a post-hoc secondary objective to report test positivity, categorizing these as proxy general population samples. Data were tabulated by population group (classified as ‘general population’, MSM and sex workers) and summarized per WHO region and country. ‘General population’ samples were identified according to WHO recommendations, to include studies conducted ‘among pregnant women, women attending family planning clinics, male military recruits and men undergoing employment physicals’ [3]. These samples served as proxies for the general population where population-based sampling was not, or could not, be conducted. The point estimates reported were adjusted for diagnostic test performance by applying a standardization factor for urogenital infection as utilized by WHO (Additional file 4) [3, 11]. For rectal and pharyngeal infections, a separate literature review was undertaken to derive sensitivity and specificity values (for culture and/or NAAT) and adjustments were applied in the same manner as for the urogenital samples (Additional file 4). Due to obvious heterogeneity in study populations and study designs, widespread inclusion of non-representative samples and frequent lack of reporting of key parameters to judge the study quality, a quality score was not assigned. Similarly, a meta-analysis could not be conducted as we were limited in our ability to appropriately compare studies directly. We did not calculate a median summary estimate per country because only a small number of countries had three or more available estimates. Instead, guided by the principles of Campbell et al [12], we conducted a narrative synthesis, presenting the prevalence and test positivity estimates reported in the context of the source population and the type of sampling conducted, rather than directly comparing estimates. General population estimates were considered ‘population-based’ and representative if participants were sampled from a general population sampling frame and some form of random selection was performed. Studies employing other forms of sampling from proxy general population samples are labelled as such. As MSM and sex workers are defined in terms of their sexual behaviour, population-based denominator samples are generally not available. For these groups, screening and/or enhanced testing is frequently recommended irrespective of symptom status (and thus may be more reflective of prevalence). Therefore, studies conducted at STI clinics and at other venues frequented by MSM and sex workers were eligible for inclusion, excepting studies including persons presenting with symptoms, which were excluded to minimize bias. The median sample size and interquartile range were estimated using Excel’s ‘quartile.exc’ function.

## Results

### Prevalence reporting in the general population

**Fig. 1:**
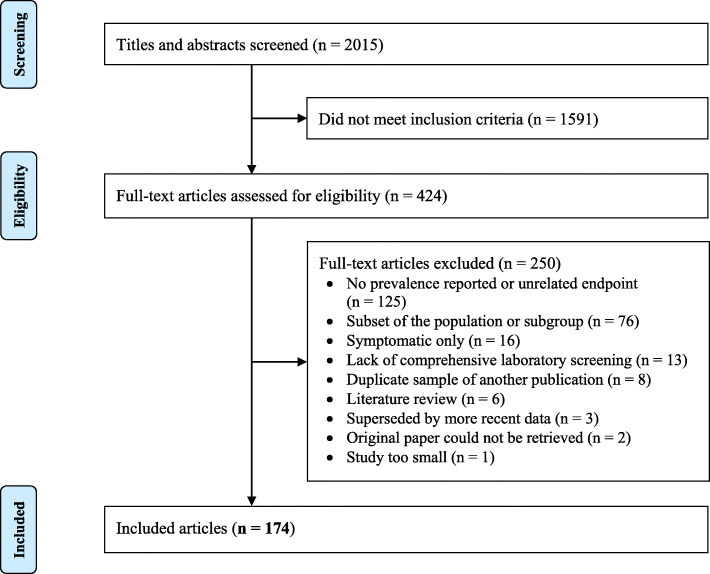
PRISMA diagram describing selection of citations reporting gonorrhoea prevalence. Note: Some articles reported outcomes on several of the populations of interest or provided data for >1 country and therefore the total number of included data points does not amount to 174. n=number of articles

**Fig. 2:**
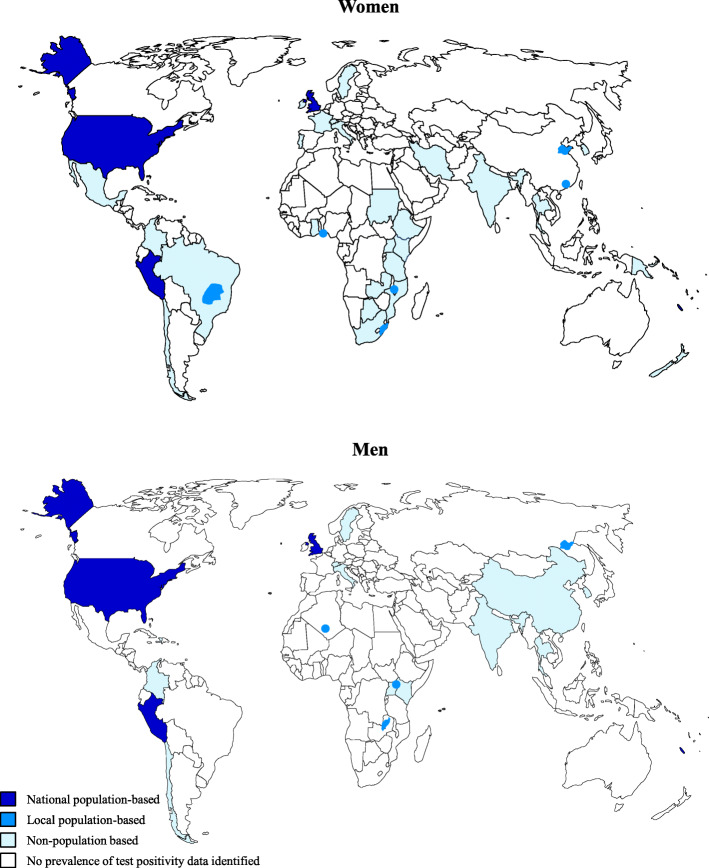
Availability of gonorrhoea prevalence reporting globally. Maps represent the availability of prevalence data in general population samples worldwide, including pregnant women, women attending family planning clinics, male military recruits, and work-based health screening programmes and other similar groups. General population estimates were considered ‘national population-based’ or ‘local population-based’ if participants were sampled from a general population sampling frame and some form of random selection was performed. Studies where probability sampling was not conducted, and which may not be generalizable beyond the study, are labelled as ‘non-population based’

We identified 2015 citations relating to gonorrhoea ‘prevalence’ (Fig. 1), subsequently categorized into (a) the general population or proxy general population groups (men, women, and pregnant women separately), (b) MSM, and (c) FSW and MSW. Following title and abstract screening, we reviewed 424 full-text publications, of which 174 addressed the primary or secondary objective and were eligible for inclusion, reporting data from the following WHO regions: Africa (n=41), the Western Pacific (n=41), high-income North America that is part of the Region of the Americas (n=25), the Americas excluding high-income North America (n=25), Europe (n=19), South-East Asia (n=18), and the Eastern Mediterranean (n=5). The number of countries where prevalence and/or test positivity estimates were identified from the general population was limited, with data points identified from only 18.0% of countries worldwide (35/194) for women and 9.8% (19/194) for men (Fig. 2). Prevalence of gonorrhoea in the general population by WHO region and country is summarized in Table 1 and test positivity estimates from proxy general population samples in Table 2.

**Table 1:** Reported population-based prevalence of gonorrhoea in women and men by WHO region and country

WHO region	Country	No. of data points	Reference	Years reported	Study population^**a**^	Study setting	Sampling	No. tested	Reported prevalence	Standardized prevalence
**WOMEN**										
Africa	Benin	1	Behanzin et al. [13]	2008	Community, Women	Households	Cluster random	1241	0.60%	0.62%
	Malawi	1	Paz-Soldan et al. [14]	2000	Community, Women	Households	Multistage cluster random	758	3.60%	4.13%
	South Africa	1	Francis et al. [15]	2016–2017	Community, Women	Annual household survey	Stratified random	259	1.80%	1.14%
Americas (excluding high-income North America)	Brazil	1	de Lima et al. [16]	2007–2009	Community, Sexually active, Women	Household-based recruitment	Simple random	574	0.70%	0.72%
	Peru^b^	1	Carcamo et al. [17]	2002	Community, Women	Household survey	Cluster random	6439	0.10%	0.00%
Europe	UK^b^	1	Sonnenberg et al. [18]	2010–2012	Community, Women	Natsal-3 respondents	Probability	2665	<0.1%	0.10%
High-income North America	USA^b^	1	Torrone et al. [19]	1999–2008	Community, Women	Household surveys	Multistage probability	··	0.34%^c^	0.37%^c^
Western Pacific	China	2	Huai et al. [20]	2016	Community, Women	General population	Multistage probability	3581	0.14%	0.14%
			Luo et al. [21]	2017	Community, Women	Community-based recruitment	Probability	9207	0.17%	0.18%
	New Caledonia (France)^b^	1	Corsenac et al. [22]	2012	Community, Women	Primary care and public dispensaries	Multistage random	376	3.47%	3.99%
**MEN**										
Africa	Benin	1	Behanzin et al. [13]	2008	Community, Sexually active, Men	Households	Cluster random	1040	0.30%	0.23%
	Malawi	1	Paz-Soldan et al. [14]	2000	Clinic attendees, Men	Households	Cluster random	469	6.20%	7.94%
	South Africa	1	Francis et al. [15]	2016–2017	Community, Men	Annual household survey	Stratified random	188	1.50%	1.82%
Americas (excluding high-income North America)	Peru^b^	1	Carcamo et al. [17]	2002	Community, Men	Household survey	Cluster random	7486	0.12%	0.02%
Europe	UK^b^	1	Sonnenberg et al. [18]	2010–2012	Community, Men	Natsal-3 respondents	Probability	1885	<0.1%	<0.1%
High-income North America	USA^b^	1	Torrone et al. [19]	1999–2008	Community, men	Household surveys	Multistage probability	··	·· (0.27% for men and women combined)^c^	·· (0.27% for men and women combined)^c^
Western Pacific	China	1	Huai et al. [20]	2016	Community, Men	General population	Multistage probability	3622	0.03%	0.00%
	New Caledonia (France)^b^	1	Corsenac et al. [22]	2012	Clinic attendees, Men	Primary care and public dispensaries	Multistage random	232	3.45%	4.36%

^**a**^To aid cross-referencing, study populations were categorized to align with SPECTRUM codes [11].

^b^Nationally derived samples.

If the standardized estimate was a negative number, the standardized prevalence was reported at 1 case divided by 100 times the sample size [11].

^c^15 885 participants aged 14–39 provided a sample. The proportion of men and women participating was not reported separately. The estimate quoted for women (0.34%) is a weighted estimate. The estimate quoted for men is that for both men and women (0.27%) as men were not reported separately. This estimate was not standardized.

··=Not reported. No.=number. UK=United Kingdom. USA=Unites States of America. WHO=World Health Organization.

**Table 2:** Reported gonorrhoea test positivity in women and men by WHO region and country

WHO region	Country	No. of data points	Reference	Years reported	Study population^**a**^	Study setting	Sampling	No. tested	Reported test positivity	Standardized estimate	
**WOMEN**											
Africa	**Proxy general population samples**										
	Ethiopia	2	Mulu et al. [23]	2013	Clinic attendees, Women	Hospital, multiple clinic types	Simple random	409	1.00%	1.25%	
			Tadesse et al. [24]	2014–2015	Ob/Gyn clinic attendees	Mixed attendees	Non-probability	322	0.31%	0.39%	
	Ghana	1	Yirenya-Tawiah et al. [25]	2005–2006	Community, Sexually active, Women	Community-based recruitment	Non-probability	191	2.60%	2.05%	
	Kenya	4	Jespers et al. [26]	2010–2011	Clinic attendees, Women	Multiple clinic types + community	Non-probability	110	1.00%	0.20%	
			Kerubo et al. [27]	2013	Students/young, Women	High school	Non-probability	511	0.59%	0.24%	
			Masese et al. [28]	2014–2015	Students/young, Women	High schools + universities	Non-probability	451	1.55%	1.60%	
			Otieno et al. [29]	2007–2009	Community, Women	Community-based recruitment	Non-probability	424	4.70%	3.99%	
	Mozambique	1	Menendez et al. [30]	2000	Mixed groups/Unknown, Women	Mixed clinic types + community	Age-stratified	250	13.60%	18.90%	
	South Africa	3	Jespers et al. [26]	2010–2011	Clinic attendees, Women	Multiple clinic types + community	Non-probability	109	1.00%	0.20%	
			Kaida et al. [31]	2014–2016	Community, Women	Community-based recruitment	Non-probability	198	7.07%	6.42%	
			Peters et al. [32]	2011–2012	Community, Women	Primary care centres	Geographic stratification	604	10.00%	10.46%	
	Uganda	2	Rassjo et al. [33]	2006	Students/young, Sexually active women	Youth clinic	Non-probability	595	4.50%	3.79%	
			Rutherford et al. [34]	2008–2009	Students/young, Sexually active women	University students	Non-probability	280	1.07%	0.69%	
	**During pregnancy**										
	Botswana	2	Offorjebe et al. [35]	2015–2016	ANC Survey	ANC	Non-probability	300	1.70%	0.92%	
			Wynn et al. [36]	2015–2016	ANC Survey	ANC	Non-probability	200	1.50%	0.72%	
	Kenya	2	Masha et al. [37]	2015	ANC Survey	ANC	Non-probability	202	1.00%	1.09%	
			Warr et al. [38]	2011–2013	ANC Survey	ANC, HIV negative women	Non-probability	1221	2.00%	1.36%	
	Sudan	2	Abdelaziz et al. [39]	2008	ANC Survey	ANC	Non-probability	200	1.80%	2.25%	
			Abdelrahim et al. [40]	··	ANC Routine screening	Pregnant women, low socioeconomic status	Non-probability	350	0.00%	0.00%	
	Tanzania	2	Chiduo et al. [41]	2008–2010	ANC Routine screening	ANC	Non-probability	185	1.62%	2.03%	
			Hokororo et al. [42]	2012	ANC Survey	ANC, adolescent girls	Non-probability	403	6.70%	7.69%	
	Zambia	1	Chaponda et al. [43]	2013–2014	ANC Survey	First time attendees	Non-probability	1083	3.14%	2.66%	
Americas (excluding high-income North America)	**Proxy general population samples**										
	Brazil	3	Piazzetta et al. [44]	··	Students/young, Sexually active women	University students (secondary analysis)	Non-probability	335	2.39%	2.47%	
			Pinto et al. [45]	2009	Clinic attendees, Women	Parturient women, national sample	Non-probability	2071	1.00%	1.09%	
			Rocha et al. [46]	2010	Community, Women	Primary healthcare	Non-probability	361	1.40%	0.68%	
	Chile	2	Conejero et al. [47]	2011	Students/young, Sexually active women	University gynaecology clinic	Non-probability	344	0.00%	0.00%	
			Huneeus et al. [48]	··	Community, Sexually active, Women	Community adolescent health clinics	Non-probability	115	0.87%	0.07%	
	Colombia	1	Paredes et al. [49]	2011	Students/young, Women	High schools	Non-probability	436	0.20%	0.22%	
	Haiti	1	Jobe et al. [50]	2012	Clinic attendees, Women	Women's health clinic	Non-probability	104	0.96%	0.18%	
	Mexico	1	Casillas-Vega et al. [51]	2013–2014	Ob/Gyn clinic attendees	Gynaecology clinic, first time attendees	Non-probability	662	2.11%	1.34%	
	**During pregnancy**										
	Brazil	1	Silveira et al. [52]	2005–2008	Clinic attendees, Women	Maternity unit, women singleton newborns	Non-probability	2101	1.14%	0.76%	
	Haiti	1	Bristow et al. [53]	2015–2016	ANC Survey	ANC	Non-probability	300	2.67%	1.91%	
Eastern Mediterranean	**During pregnancy**										
	Iran		Pourabbas et al. [54]	··	Clinic attendees, Women	Maternity unit	Non-probability	239	1.26%	0.49%	
Europe	**Proxy general population samples**										
	Ireland	1	Hassan et al. [55]	··	Community, Women	Women attending primary care for cervical screening	Non-probability	236	0.00%	0.00%	
	Italy	2	Matteelli et al. [56]	2012–2013	Students/young, Women	High school	Non-probability	1606	0.00%	0.00%	
			Salfa et al. [57]	2009–2013	Mixed groups/Unknown, Women	Lab reports from tests at multiple healthcare providers including screening tests	Geographic stratification	40 579	0.1%	0.1%	
	Sweden	1	Nolskog et al. [58]	2013–2014	Students/young, Women	Youth clinics	Systematic	509	0.00%	0.00%	
	Switzerland	1	Sakem et al. [59]	2009–2010	Mixed groups/Unknown, Women	Laboratory samples including screening programmes	National laboratory database	8009	0.14%	0.00%	
	UK	1	Grech et al. [60]	2014–2015	Clinic attendees, Women	Integrated sexual health service, women >40 years	Systematic	150	0.70%	0.01%	
	**During pregnancy**										
	France	1	Peuchant et al. [61]	2011	ANC Routine screening	ANC	Non-probability	1004	0.00%	0.00%	
	Portugal	1	Borges-Costa et al. [62]	2006–2008	Clinic attendees, Women	Pregnant adolescents attending obstetric hospital clinic	Non-probability	204	4.90%	4.90%	
High-income North America	**Proxy general population samples**										
	USA	4	CDC (National Job Training Program) [63]	2018	Community, women	National Job Training Program for socioeconomically disadvantaged youth	Non-probability	··	2.2%^b^	2.2%^b^	
			Jackson et al. [64]	2009–2010	Clinic attendees, Women	Chart review of women > 25 years screened according to guidelines in Baltimore	Non-probability sample of consecutive attendees	658	0.30%	0.00%	
			Newbern et al. [65]	2003–2010	Students/young women	Participants in Philadelphia high school STI screening programme	Non-probability	36 263	9.00%^c^	9.32%^c^	
			Nsuami et al. [66]	2003–2005	Students/young women	Participants in New Orleans high school STI screening programme	Non-probability	1554	·· (2.4% for men and women combined)^d^	·· (2.4% for men and women combined)^d^	
	**During pregnancy**										
	USA	4	Akoh et al. [67]	2006–2009	ANC Survey	Adolescent maternal programme	Stratified by ethnicity/race	158	3.00%	3.00%	
			Berggren et al. [68]	2003–2005	ANC Survey	Washington Hospital Center	Non-probability	125	10.00%	10.00%	
			Blatt et al. [69]	2005–2008	Pregnant women, Community	Laboratory results from pregnant women	Database study	730 796	0.63%	0.63%	
			Waight et al. [70]	2007–2009	Mixed groups/Unknown, Women	STD diagnoses linked to birth records	Database study	195 977	1.55%	1.55%	
South-East Asia	**Proxy general population samples**										
	India	1	Krishnan et al .[71]	Not stated	Community, Women	Samples collected during household visits	Non-probability	811	0.00%	0.00%	
	**During pregnancy**										
	Thailand	1	Asavapiriyanont et al. [72]	2006–2007	ANC Survey	Pregnant teenagers	Non-probability	121	1.70%	1.76%	
Western Pacific	**Proxy general population samples**										
	Korea (Rep. of)	2	Choe et al. [73]	2010	Community, Women	General population health examination centres	Non-probability	805	0.25%	0.26%	
			Kim et al. [74]	2012	Community, Sexually active, Women	Hospital clinics, health women attending for checkups	Non-probability	799	0.00%	0.00%	
	Papua New Guinea	1	Vallely et al. [75]	2011–2015	Community, Women	Well woman clinic only	Non-probability	614	7.98%	8.17%	
	Solomon Islands	1	Marks et al. [76]	2014	Clinic attendees, Women	Community outpatient clinics	Non-probability	296	5.10%	4.40%	
	**During pregnancy**										
	New Zealand	1	Ekeroma et al. [77]	2009	ANC Routine screening	Hospital maternity unit	Non-probability	4635	0.22%	0.20%	
	Papua New Guinea	3	Badman et al. [78]	2014	ANC Routine screening	Women at first antenatal visit	Non-probability	125	11.20%	10.65%	
			Vallely et al. [75]	2011–2015	ANC Survey	ANC	Non-probabilitynon-probability	765	14.20%	15.24%	
			Wangnapi et al. [79]	2011–2012	ANC Survey	ANC	Non-probability	362	9.67%	9.08%	
**MEN**											
Africa	Kenya	1	Otieno et al. [29]	2007–2009	Community, Sexually active, Men	Community-based recruitment	Non-probability	422	0.00%	0.00%	
	South Africa	1	Kaida et al. [31]	2014–2016	Community, Men	Community-based recruitment	Non-probability	154	1.30%	1.40%	
	Uganda	1	Rutherford et al. [34]	2008–2009	Students/young, Men	University students	Non-probability	360	0.00%	0.00%	
Americas (excluding high-income North America)	Chile	1	Huneeus et al. [48]	··	Community, Sexually active, Men	Community adolescent health clinics	Non-probability	171	0.00%	0.00%	
	Colombia	1	Paredes et al. [49]	2011	Students/young, Men	High schools	Non-probability	536	0.00%	0.00%	
	Haiti	1	Downey et al. [80]	2013	Clinic attendees, Men	Men's health clinic	Non-probability	205	0.00%	0.00%	
Europe	Italy	2	Matteelli et al. [56]	2012–2013	Students/young, Men	High school	Non-probability	1112	0.00%	0.00%	
			Salfa et al. [57]	2009–2013	Mixed groups/Unknown, Men	Lab reports from tests at multiple healthcare providers	Geographic stratification	10 243	2.10%	2.10%	
	Sweden	1	Nolskog et al. [58]	2013–2014	Students/young, Men	Youth clinics	Systematic	492	0.20%	0.12%	
	Switzerland	1	Sakem et al. [59]	2009–2010	Mixed groups/Unknown, Men	Laboratory samples incl. screening programmes	National laboratory database	1236	2.10%	1.82%	
High-income North America	USA	4	CDC (National Job Training Program) [63]	2018	Community, Men	National Job Training Program for socioeconomically disadvantaged youth	Non-probability	··	0.7%^b^	0.7%^b^	
			Drinkard et al. [81]	2009–2015	Students/young, Men	College health clinics	Database study	5453	0.70%	0.22%	
			Newbern et al. [65]	2003–2010	Students/young men	Participants in Philadelphia high school STI screening programme	Non-probability	39 010	4.07%^c^	4.65%^c^	
			Nsuami et al. [66]	2003–2005	Students/young men	Participants in New Orleans high school STI screening programme	Non-probability sample of participants	1782	·· (2.4% for men and women combined)^d^	·· (2.4% for men and women combined)^d^	
South-East Asia	India	1	Dave et al. [82]	2005	Workers, Men	Migrant workers	Multistage probability	840	0.90%	0.94%	
	Thailand	1	Jatapai et al. [83]	2008–2009	Military, Men	Newly inducted military conscripts	Systematic	2123	0.94%	1.04%	
Western Pacific	China	1	Zhang et al. [84]	2006	Workers, Men	Miners	Cluster	1773	0.81%	0.81%	
	Korea (Rep. of)	1	Choe et al. [73]	2010	Clinic attendees, Men	General population health examination centres	Non-probability	807	0.62%	0.61%	

^**a**^To aid cross-referencing, study populations were categorized to align with SPECTRUM codes [11].

If the standardized estimate was a negative number, the standardized prevalence was reported at 1 case divided by 100 times the sample size [11].

If neither the clinical specimen nor the laboratory test used were specified, the estimate was not standardized [57, 62, 63, 67–70, 84].

If the clinical specimen was specified but not the laboratory test used, or vice versa, the arithmetic mean of the sensitivity and specificity for the laboratory test or for the clinical specimen, respectively, was used instead [27, 34, 52, 59, 80, 81].

^b^The denominator was not reported and estimates were not standardized.

^c^Prevalence was reported over an 8-year period.

^d^Prevalence proportion for men and women was not reported separately.

··=Not reported. ANC=antenatal clinic. CDC=Centers for Disease Control and Prevention. HIV=human immunodeficiency virus. No.=number. Ob/Gyn=Obstetrics and Gynaecology. Rep.=Republic. STD=sexually transmitted disease. STI=sexually transmitted infection. UK=United Kingdom. USA=Unites States of America. WHO=World Health Organization.

For several countries, we did not identify prevalence or test positivity data. The grey literature search led to one additional estimate [63], but also allowed us to set the prevalence estimates identified in the context of the extent of surveillance otherwise ongoing in the country. To this end, expert consultation led to identification of surveillance data from three international reporting networks (WHO Global, WHO European Regional Office, and ECDC), and national surveillance data or reports from an additional seven countries, the United States of America (USA), Canada, Australia, Singapore, New Zealand, Japan, and the Republic of Korea.

### Europe

Prevalence data and/or test positivity in general population samples were identified in 13.2% (seven out of 53) of countries in the WHO European region (nine estimates in women, including pregnant women [18, 55–62], and five in men [18, 56–59]) (Tables 1 and 2). We identified only one representative, population-based prevalence study in the United Kingdom (UK) that was of national scope [18]. These data were derived from the National Survey of Sexual Attitudes and Lifestyles (NATSAL) in 2010–2012. A probability sample of 15 162 men and women aged 16–74 years was drawn from the general population. Gonorrhoea testing was conducted for 2665 women and 1885 men and an overall prevalence of <0.1% was recorded (Table 1), higher in women and men aged 20–24 (0.2% and 0.1%, respectively). Data from all other countries represented test positivity data that were drawn from proxy groups of the general population, mainly non-probability samples, drawn from antenatal/obstetric clinics, primary care, community/youth clinics, with one study in a high school setting [56]. The median study sample size was 1004 in all women (interquartile range [IQR]: 220–5337) and 1236 in men (IQR: 802–6620). In all general population studies, NAAT testing conducted on urine (men, women) or genital fluid (women) was most common; confirmation by both NAAT and culture was used in pregnant women in France and Portugal [61, 62]. Data on both sexes were available in only five studies [18, 56–59]. One study reported samples from the urogenital and rectal site in aggregate [57]. All other studies included urogenital infection only.

For countries where no prevalence or test positivity estimate from the general population was identified, some degree of surveillance data was discoverable through the grey literature search. Most European Union (EU)/European Economic Area (EEA) Member States have comprehensive surveillance systems and report a national notification rate annually, except for Germany, Liechtenstein, Austria (not since 2014), and Greece (not since 2017) [85, 86]. Belgium, France, and the Netherlands have sentinel surveillance systems. In countries outside the EU/EAA region (mostly the eastern European region), data were less discoverable. In 2017, countries including Armenia, Azerbaijan, Belarus, Georgia, Kazakhstan, Kyrgyzstan, Russian Federation, Turkmenistan, and Uzbekistan reported gonorrhoea cases to the WHO European Regional Office (M. Dara and G. Kuchukhidze, personal communication, 24 February 2019). Indicators included the absolute number of cases identified, the male to female ratio, and only for Armenia, the proportion of reported MSM among the cases. Prevalence data or comprehensive syndromic and aetiologic case reporting were not otherwise identified in the wider European region.

### High-income North America

In the USA, laboratory-confirmed gonorrhoea is mandatorily notifiable and data collection is comprehensive, from diverse clinical settings including STD clinics, laboratories, family planning and school-based clinics, hospitals, emergency rooms, drug treatment centres, correctional facilities, and the military [87]. The most recent estimate of nationwide population prevalence identified was from the National Health and Nutrition Examination Survey (NHANES), a series of cross-sectional, bi-annual household surveys representative in terms of sex, age and race/ethnicity of the USA civilian, non-institutionalized population [19]. Between 1999 and 2008, screening for cervical or urethral gonorrhoea was a study component, and 15 885 persons, aged 14–39 years participated. An extrapolated national prevalence of 0.3% (95% confidence interval [CI]: 0.1%–0.5%) among 14–39-year-olds was estimated, higher in women than in men (Table 1). *N. gonorrhoeae* testing within NHANES stopped at the end of 2008 and, in 2009, gonorrhoea prevalence and notification rates were at an all-time low in the USA [19].

In terms of non-probability samples, an estimate of prevalence from a sentinel surveillance population of young people at elevated risk for gonorrhoea is provided annually by the Centers for Disease Control and Prevention (CDC), using data from the ‘National Job Training Program’ (NJTP), a nationwide vocational programme for socioeconomically disadvantaged youth aged 16 to 24 years who are considered at risk of STIs [63]. Participants are offered gonorrhoea and chlamydia screening at programme entry. In 2018, the median state-specific estimated gonorrhoea prevalence for programme entrants aged 16–24 years was 2.2% in women (range 0.4% to 7.6%), and 0.7% in men (range 0.0% to 4.8%) (Table 2) [63].

In the USA, we identified a further seven test positivity estimates from proxy general population samples in women (including one from a chart review of women screened [64], two studies in high schools [65, 66], and four in pregnant women who are routinely tested [67–70]) and three data points in men (the same two studies in high schools [65, 66] and one study in college students [81]) that met the inclusion criteria for the secondary objective (Table 2). There was a wide range in study sample size and in estimates reported, reflecting diversity in study participants and settings, and study population characteristics. Test positivity estimates from non-probability samples from the two studies in high schools were identified: one reported the proportion positive over almost 8 years (9.0% [3270/36 263] in girls and 4.1% [1588/39 010] in boys) and another yielded a combined estimate of 2.4% in girls and boys (Table 2) [65, 66]. No comparison could be made across studies. Where reported, studies used NAAT testing.

In Canada, no prevalence study or proxy general population study was identified. Gonorrhoea is mandatorily notifiable, and laboratory-confirmed cases are reported to the Public Health Agency of Canada through the Canadian Notifiable Disease Surveillance System. Summary data are published annually by age and sex, and are available online [88], and a detailed surveillance report is produced every five years.

### Americas (excluding high-income North America)

Prevalence and/or test positivity estimates from the general population were identified in 18.2% (six out of 33) of countries in this WHO region excluding the USA and Canada (12 estimates in women, including pregnant women [16, 17, 44–53], and four in men [17, 48, 49, 80]) (Tables 1 and 2). One study in Peru could be considered population-based and of national scope. In this study, the substantial sample included 13 925 randomly selected 18–29-year-old men and women who were resident in 24 cities with populations >50 000 people [17]. Additionally, a local population-based study in Brazil, also urban, was conducted using two-stage sampling of households and young women in middle size cities in Central Brazil [16]. The remaining studies were non-probability samples, mainly from community settings including educational facilities, primary healthcare, adolescent health clinics and ANCs. The median study sample size was 399 in women (IQR: 309-1719) and 371 in men (IQR: 180-5749). All studies involved NAAT screening of urine (n=4) [16, 44, 45, 49] and urogenital swab samples (n=7) [17, 46–48, 50, 51, 53] for women (clinical specimen not specified, n=1 [52]), and urine (n=3) [17, 48, 49] for men (clinical specimen not specified, n=1 [80]).

From the grey literature search, we identified only aetiological or syndromic case reporting in adult men through WHO Global AIDS Monitoring (GAM; known as Global AIDS Response Progress Reporting prior to 2015) for other countries in the region [3]. No further prevalence or test positivity data were identified in the region.

### Africa

In the WHO African region, prevalence data and/or test positivity estimates from the general population were identified in 25.5% (12 out of 47) of countries (25 estimates in women, including pregnant women [13–15, 23–43], and six in men [13–15, 29, 31, 34]) (Tables 1 and 2). Three of the studies were local population-based, derived from household samples, and none were of national scope. The first was from the urban centre of Cotonou in Benin, where 2507 subjects aged 15–49 years, from 1070 households sampled from 38 census areas, participated [13]. In Malawi, another estimate was derived from a largely rural population from the eastern lakeside regions of the Mangochi district [14]. Most recently, 1342 young people aged 15–24 years were selected from a ‘health and demographic surveillance site’ sampling frame in rural South Africa [15]. The remaining studies (Table 2) were derived from non-probability samples with diverse recruitment sites, including antenatal clinic (ANC) settings, schools and universities, primary healthcare sites, and community-based recruitment. The median study sample size was 322 in women (IQR: 200–553) and 422 in men (IQR: 351–755). Laboratory confirmation was mainly by NAAT on urogenital swab samples and, to a lesser extent, on urine for women; in four studies [23, 24, 30, 40], Gram stain and/or culture only were used. For men, urine samples were tested by NAAT in all cases where reported.

In the African region, 43% of countries reported to WHO in 2013 having STI surveillance systems in place and 40% had national strategies or plans for preventing and controlling STIs [89], but beyond limited reporting of aetiological surveillance among men and syndromic surveillance in men and women, we did not identify any further prevalence reporting in the region.

### Western Pacific

Prevalence data and/or test positivity data from the general population were identified in 22.2% (six out of 27) of countries and territories in the WHO Western Pacific region (11 estimates in women, including pregnant women [20–22, 73–79], and four in men [20, 22, 73, 84]) (Tables 1 and 2). There were three population-based studies. One was of national scope in New Caledonia [22]. It included men and women selected during a national three-stage random sampling of general practice surgeries and public dispensaries, and the sample was then weighted to reflect the general population aged 18–49 years. The other two population-based studies were local in scope and were both in China. In one study from the Shandong province [20], men and women were sampled in a complex multi-stage sampling process based on urban and rural communities within geographic regions. The second study, from Shenzhen City [21], included women only and was designed to be representative of the entire population in the Nanshan District of the city. Beyond these prevalence data, for both men and women, test positivity estimates from non-probability, proxy general population samples were derived from a range of study settings including community settings, primary care, and hospital-based maternity clinics/ANCs. One study was in an occupational group (miners) in men in China [84]. The median study sample size in the region was 765 in women (IQR: 362–3581) and 1290 in men (IQR: 376–4490). In some countries in the region where no data were identified, gonorrhoea is a notifiable infection; routine national surveillance is conducted and opportunistic/risk-based screening and/or testing is recommended for some population groups (Australia, New Zealand, and Singapore) [90–92]. Sentinel surveillance is conducted in the Republic of Korea and in Japan, mostly in urology departments. In both countries, reported cases per sentinel are low and have decreased in recent years [93, 94]. GAM data for men are also notified to WHO from many countries [3], but no further prevalence data were identified in the region.

### South-East Asia

Among 11 countries in the WHO South-East Asian region, we did not identify any population-based prevalence estimates. Test positivity data in general population samples were identified from 18.2% (two out of eleven) of countries (two estimates in women, including pregnant women [71, 72], and two in men [82, 83]) (Table 2). Non-probability samples from the general population were community-based in women in India, hospital-based in young pregnant women aged <18 years in Thailand, and in occupational groups in men (migrant workers in India and military conscripts in Thailand). The median study sample size was 466 in women (range: 121–811) and 1482 in men (range: 840–2123).

We did not identify further information on gonorrhoea surveillance in the region, with the exception of GAM data from some countries [3].

### Eastern Mediterranean

We identified non-probability samples in 4.8% (one out of 21) of countries in the WHO Eastern Mediterranean region: a single study in a hospital involving pregnant women [54] (Iran, n=239; standardized prevalence: 0.5%) (Table 2). According to the WHO, ten countries surveyed in 2013 reported having an STI surveillance system, four reported conducting aetiological studies, 11 had updated national STI guidelines or recommendations in place and nine had a national strategy or action plan for STI prevention and control [89], but no further estimates were identified in the region.

### Prevalence and test positivity reporting in vulnerable population groups

#### Men-who-have-sex-with-men

**Fig. 3:**
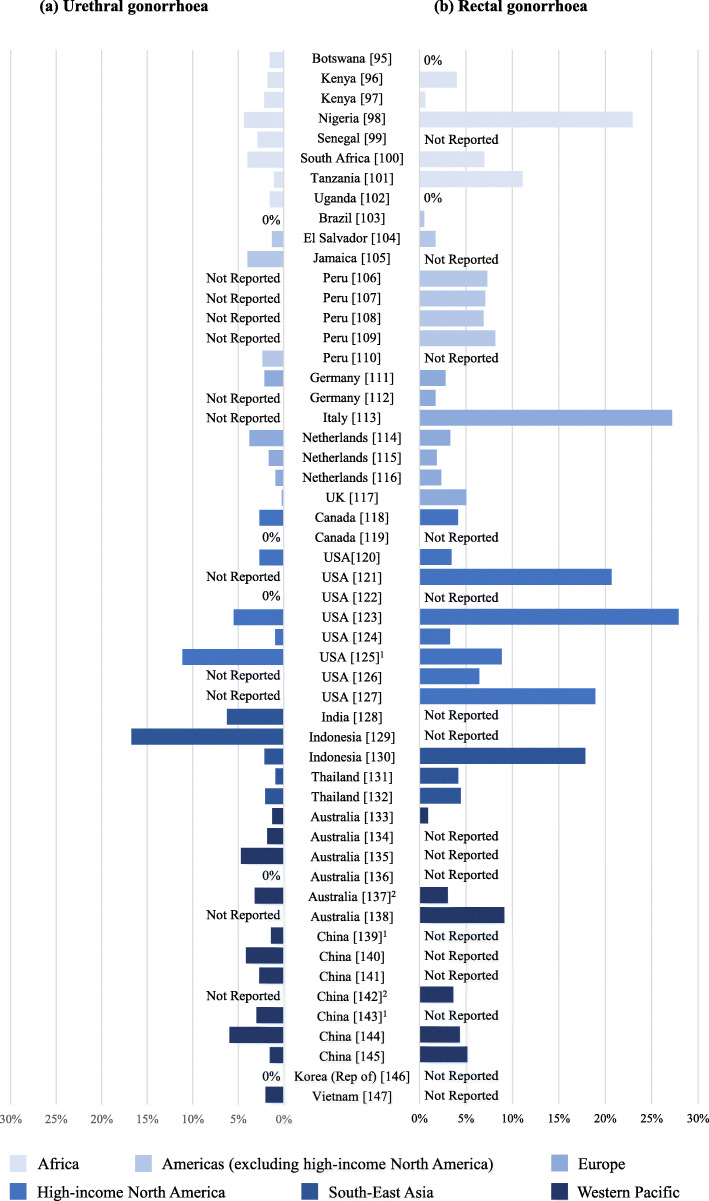
Reported prevalence and/or test positivity of urethral (a) and/or rectal (b) gonorrhoea-positive cases in men-who-have-sex-with-men. ^1^Prevalence rates for urethral gonorrhoea could not be standardized. ^2^Prevalence rates for rectal gonorrhoea could not be standardized

Prevalence and/or non-probability test positivity data on gonorrhoea in the MSM population were identified in 64 studies from 25 countries (seven countries in Africa, five in Europe, two in North America, four in the Americas [excluding high-income North America], four in the Western Pacific, and three in South-East Asia) (Fig. 3; Additional file 5) [95–147]. For 56.0% (14 out of 25) of countries, data originated from a single study in an urban setting. In five studies, men testing HIV-positive were excluded at the outset [96, 119, 124, 148, 149]. HIV status was reported in five studies with variable HIV-positivity [98, 100, 103, 115, 127]. Three studies included asymptomatic cases only [120, 131, 150]. Urogenital screening and/or opportunistic testing (predominantly on urine samples) was most often performed. An equal proportion of studies involved recruitment from community settings or STI clinics, but there was diversity in terms of the populations included, including HIV status, which was often not reported. Both rectal and urogenital sampling were reported in 26 studies; rates of rectal infection were higher than urogenital rates in 69.2% (n=18) of these studies (Fig. 3). NAAT testing was reported in 22 of these studies, culture-only testing in two, and culture or NAAT testing in two. Reported rates of pharyngeal testing from 27 studies were mostly (51.9%) between 5.0% and 10.0%, 22.2% were between 1.0% and 5.0%, and 14.8% were >10.0%. Though variable, on average, the standardized estimate of pharyngeal infection was similar to urogenital positivity where reported in the same study.

#### Sex workers

Data on gonorrhoea prevalence and/or test positivity in MSW, FSW or both were available from 23 countries (Table 3), with 38 studies reporting on FSW and six on MSW. Of 41 unique studies, 14 were conducted in a clinic setting (including STI clinics, genito-urinary clinics and outreach clinics) and 13 at commercial sites (including hotels, brothels, street and residence). The remainder (n=14) were described as community-based or conducted at other mixed locations. Only urogenital testing was performed except for one study in China that also performed pharyngeal testing [184]. Overall, the median study sample size was 655 in women (IQR: 323–2165) and 240 in men (IQR: 113–584). The positivity estimates ranged from 0.0% (MSW in the Republic of Korea) to 29.2% (FSW in Indonesia).

**Table 3:** Reported gonorrhoea prevalence and/or test positivity in sex workers, by WHO region and country

WHO region	Country	No. of data points	Reference	Years reported	Study setting^**a**^	No. tested	Reported prevalence and/or test positivity	Standardized estimate
**WOMEN**								
Africa	Benin	1	Behanzin et al. [151]	2008	Clinic setting	1082	6.20%	5.53%
	Botswana	1	Merrigan et al. [152]	2012	Community-based or other mixed locations	947	10.50%	10.86%
	Cote d'Ivoire	1	Vuylsteke et al. [153]	2007 and 2009	Clinic setting	1110	5.10%	4.40%
	Ethiopia	1	Tadele et al. [154]	2017	Clinic setting	338	3.30%	4.13%
	Guinea	1	Aho et al. [155]	2005–2006	Clinic setting	223	9.00%	8.40%
	Kenya	1	Izulla et al. [156]	2009–2010	Clinic setting	2933	3.07%	3.84%
	Rwanda	2	Braunstein et al. [157]	2006–2007	Clinic setting	397	11.60%	11.06%
			Jespers et al. [26]	2010–2011	Community-based or other mixed locations	30^b^	7.00%	6.35%
	Uganda	1	Vandepitte et al. [158]	2008–2009	Community-based or other mixed locations	1025	13.00%	12.49%
Americas (excluding high-income North America)	Guatemala	1	Sabido et al. [159]	2008–2009	Commercial site	494	0.80%	0.83%
	Honduras	1	Tinajeros et al. [160]	2006–2008	Community-based or other mixed locations	950	2.30%	1.54%
	Mexico	1	Bazzi et al. [161]	2010–2013	Community-based or other mixed locations	212	0.94%	0.98%
	Peru	1	Carcamo et al. [17]	2002	Commercial site	4263	1.62%	1.25%
Eastern Mediterranean	Iran	2	Kazerooni et al. [162]	2010	Community-based or other mixed locations	278	1.43%	1.79%
			Nasirian et al. [163]	2013–2014	Community-based or other mixed locations	99^b^	9.09%	8.49%
	Pakistan	1	Khan et al. [164]	2007	Commercial site	730	7.50%	6.86%
	Tunisia^c^	1	Znazen et al. [165]	2007	Clinic setting	188	3.72%	4.66%
		1	Znazen et al. [165]	2007	Clinic setting	188	11.17%	10.62%
Europe	UK	1	Mc Grath-Lone et al. [166]	2011	Clinic setting	2534	2.70%	1.95%
South-East Asia	Bangladesh	2	Haseen et al. [167]	2006–2007	Commercial site	1013	2.20%	2.27%
			Khanam et al. [168]	2014	Commercial site	700	5.40%	4.71%
	India	2	Das et al. [169]	2008–2008	Clinic setting	417	14.20%	13.72%
			Hemalatha et al. [170]	2005–2006	Community-based or other mixed locations	3223	1.99%	2.05%
	Indonesia	5	Bollen et al. [171]	2008–2009	Clinic setting	580	29.31%	29.19%
			Majid et al. [172]	2006–2007	Commercial site	4324	24.60%	24.37%
			Mawu et al. [173]	2008	Commercial site	217	10.60%	10.03%
			Silitonga et al. [174]	1997–2002	Clinic setting	3073	16.69%	20.88%
			Tanudyaya et al. [175]	2005	Community-based or other mixed locations	2500	28.60%	28.46%
Western Pacific	Cambodia	1	Couture et al. [176]	2007–2008	Community-based or other mixed locations	160	7.80%	8.06%
	China	9	Chen et al. [177]	2009	Commercial site	3099	5.91%	5.23%
			Guo et al. [178]	2010–2011	Community-based or other mixed locations	609	2.30%	1.54%
			Jin et al. [179]	2008	Commercial site	568	8.30%	7.68%
			Luo et al. [180]	2009–2012	Commercial site	2053	8.00%	7.37%
			Remis et al. [181]	2009	Commercial site	750	3.50%	3.62%
			Tang et al. [182]	2009	Commercial site	849	5.42%	4.73%
			Wong et al. [183]	2007	Clinic setting	503	1.79%	1.01%
			Wong et al. [184]	2012–2013	Community-based or other mixed locations	340	0.90%	0.93%
			Zhu et al. [185]	2007	Commercial site	488	1.84%	1.06%
**MEN**								
Africa	Cote d'Ivoire	1	Vuylsteke et al. [186]	2007–2008	Clinic setting	96^b^	12.80%	11.75%
Americas (excluding high-income North America)	Mexico	2	Bazzi et al. [161]	2010–2013	Community-based or other mixed locations	212^d^	1.42%	1.54%
			Galarraga et al. [187]	··	Clinic setting	267	2.26%	2.53%
Europe	UK	1	Mc Grath-Lone et al. [166]	2011	Clinic setting	447	17.40%^e^	16.33%
Western Pacific	Korea (Rep. of)	1	Jung et al. [146]	2008	Community-based or other mixed locations	118	0.00%	0.01%
	Vietnam	1	Goldsamt et al. [188]	2014–2016	Community-based or other mixed locations	995	10.45%	9.41%

^a^Clinic settings included STI clinics, genito-urinary clinics and outreach clinics. Commercial sites included hotels, brothels, street and other residences. The remainder were community-based or conducted at other mixed locations, or the location was not specified.

If the standardized estimate was a negative number, the standardized prevalence was reported at 1 case divided by 100 times the sample size [11].

If the clinical specimen was specified but not the laboratory test used, or vice versa, the arithmetic mean of the sensitivity and specificity for the laboratory test or for the clinical specimen, respectively, was used instead [17].

^b^Given the rarity of data, this study was included despite a sample size of <100.

^c^In this study, two separate estimates were generated in the same population – 188 participants were tested by culture and the same 188 participants by NAAT.

^d^Men included in this study are non-commercial, intimate partners of female sex workers.

^e^This is a period prevalence, defined as the proportion of individuals tested for a sexually transmitted infection in 2011 who experienced an episode of that infection.

··=Not reported. NAAT=nucleic acid amplification test. No=number. UK=United Kingdom. WHO=World Health Organization.

## Discussion

Gonorrhoea prevalence monitoring is one of four key components of national STI surveillance programmes that is recommended by WHO to reduce the burden of gonorrhoea infections by 90% between 2018 and 2030 (in addition to case reporting, assessment of the aetiology of STI syndromes, and monitoring of antimicrobial resistance) [9]. WHO recommends prevalence assessments in the general population every two to three years, and in key populations such as MSM and sex workers [3, 9]. From our review, it is clear that substantive prevalence data among representative samples of the general population were seriously lacking on a worldwide basis. We identified national population-based data from only four countries (USA [19], UK [18], Peru [17], and New Caledonia [22]), all pre-dating 2013. Recent local population-based data were identified from China [20, 21] (2016 and 2017) and South Africa [15] (2018), but otherwise samples used for local population-based estimates were collected more than 10 years ago (Brazil, Benin, and Malawi [13, 14, 16]). The majority of the remaining test positivity estimates were derived from non-probability samples from groups that might be considered proxies of the general population, as proposed by WHO [3].

Based on our findings, most studies were conducted in single centres or discrete geographic regions or populations. We excluded STI clinic settings to avoid overestimating the prevalence in general population samples. As estimates (mainly from proxy general population groups) tended to be high, albeit with wide variation in the magnitude and precision of the estimate, it is highly likely that the risk profile of proxy populations was also higher than that of the general population. Even within groups, representativeness may not always have been similar (e.g. military conscripts residing in barracks versus those living at home). The median study sample size in the Americas (excluding high-income North America), Africa and the Eastern Mediterranean was <500 in both men and women, which further limits generalizability beyond the study population in question. Where national data were available to comment (e.g. USA), estimates from non-probability samples in defined younger populations [63, 65, 66] were higher than nationwide population-based estimates [19] or estimates from older populations [64], further highlighting the need for continued nationally representative population sampling. Diagnostic testing used varied widely and the sensitivity and specificity of these are an essential factor, contributing to differences in reported estimates. We standardized estimates for differences in laboratory methods (NAAT versus culture) and clinical specimens (urine or urogenital samples) where reported [3, 11]. For consistency, we also adjusted for NAAT versus culture on rectal and pharyngeal samples, based on reported sensitivities and specificities in the literature and using a similar standardization procedure, to allow for within-study comparison. The specimen and test were not always reported, but NAAT-based testing was most common.

For most countries, no prevalence estimate or test positivity estimate from general population groups was identified. It was clear from our online (English) grey literature search, that surveillance is ongoing more widely, as we retrieved surveillance reports from online national and international data repositories, syndromic surveillance reports in some countries, and intermittent summaries of laboratory surveillance in others. Generally, the quality and quantity of data identified were highly variable and often neither timely nor contemporaneous. In the absence of prevalence data, low case rates reported in some settings likely reflect limited testing and restricted availability of appropriate laboratory diagnostics rather than actual infection rates. In many African countries, for example, prevalence reports (where available) and syndromic surveillance suggest that the very limited aetiological reporting substantially underestimates the true infection burden.

Heterogenous data in MSM were available for only 12.9% (25 out of 194) of countries, mainly single-centre studies in urban, community-based or STI clinic settings. Most studies performed testing at the urogenital site. Where both urogenital and extragenital testing were conducted, rates at rectal sites were typically higher. However, for modern NAATs no evidence-based consensus exists regarding sensitivity and specificity correction factors when using other diagnostic methods or different NAATs for urogenital and especially extragenital infections. International evidence-based consensus regarding these corrections is imperative to develop. Rates among FSW were often many multiples higher than general population estimates in women, in countries where data in both populations were available. Due to the dearth of data on sex workers in some regions, we erred on the side of inclusivity, including small studies of <100 from Iran for example, where no data were otherwise available.

Our review had limitations. There were undoubtedly data from studies not discoverable on PubMed. For example, a systematic review from China, which documented STI risk among MSM [189], included studies that we could not access through the library systems available to us. Systematic reviews on a regional basis with good local knowledge, including in setting and language, would be a valuable addition. For many countries, only one or two data points were identified. Limited data and marked heterogeneity between studies prohibited us from conducting a meta-analysis or reporting median estimates. Reporting of proportion testing positive was very diverse in terms of variables reported, degree of stratification by demographic and other factors (e.g. HIV status), details regarding diagnostic tests, and anatomic site, often with statistics omitted where data had clearly been collected. With improved reporting from diverse populations, novel methods for synthesizing diverse data may therefore be required.

## Conclusions

Gonorrhoea prevalence is a core indicator to properly inform gonorrhoea management and control programmes, international and national guidelines, and policy documents. Gonorrhoea prevalence monitoring and reporting is suboptimal or absent in most countries. Many countries and regions have seen substantial increases in notification rates of gonorrhoea in recent years [63, 85]. In the absence of serial prevalence data, however, it is difficult to disentangle how much of this reflects a true increase in the burden of gonorrhoea or some degree of improved awareness among groups at increased risk (in particular MSM), more consistent screening and/or testing, increased availability and use of NAATs, and improved (electronic) reporting. Irrespectively, among key populations such as MSM and sex workers, there is a substantial burden of infection where data are available. To inform STI control programmes at the national and regional level, and to inform innovative epidemiologic modelling initiatives such as SPECTRUM [11] and the Global Burden of Disease [190] that attempt to quantify and model the global burden, significantly more data of higher quality are required. There is an urgent need for more resources for researchers to design, conduct and report prevalence studies in a more consistent, standardized, and quality-assured way. Within countries, serial prevalence monitoring at intervals, including assessment and reporting of a minimum set of epidemiological variables, should be considered. Our review showed the need for more testing at extragenital sites, particularly, but not exclusively, among the MSM population. WHO currently provides guidance on the assessment of gonorrhoea and chlamydia prevalence among pregnant women at ANCs [191]. This guidance could be extended beyond the ANC setting. Consistent adherence to study reporting guidelines (e.g. adapted STROBE checklists [192] or equivalent), for all researchers is also advised.

## Supplementary Information

**Additional file 1.** PRISMA checklist.

**Additional file 2.** Literature search strategy.

**Additional file3.** Inclusion and exclusion criteria.

**Additional file 4.** Standardizations adopted for urogenital, rectal and pharyngeal laboratory tests.

**Additional file 5.** Reported gonorrhoea prevalence and/or test positivity in men-who-have-sex-with-men, by WHO region, country, and anatomic site.

**Additional file 6.** Dataset supporting the output of the literature search.

## Data Availability

The dataset supporting the conclusions of this article is included within the article and its additional file (Additional file 6).

## Abbreviations

AMR: Antimicrobial resistance
ANC: Antenatal clinic
CDC: Centers for Disease Control and Prevention
CI: Confidence interval
ECDC: European Centre for Disease Prevention and Control
EEA: European Economic Area
EU: European Union
FSW: Female sex workers
GAM: Global AIDS Monitoring
HIV: Human immunodeficiency virus
IQR: Interquartile range
MeSH: Medical subject headings
MSM: Men-who-have-sex-with-men
MSW: Male sex workers
NAATs: Nucleic acid amplification tests
NATSAL: National Survey of Sexual Attitudes and Lifestyles
NHANES: National Health and Nutrition Examination Survey
NJTP: National Job Training Program
STD: Sexually transmitted disease
STI: Sexually transmitted infection
UK: United Kingdom
USA: United States of America
WHO: World Health Organization

## References

[1] Rowley J, Vander Hoorn S, Korenromp E, Low N, Unemo M, Abu-Raddad LJ (2019). Chlamydia, gonorrhoea, trichomoniasis and syphilis: global prevalence and incidence estimates, 2016. Bull World Health Organ..

[2] Unemo M, Seifert HS, Hook EW, Hawkes S, Ndowa F, Dillon JR (2019). Gonorrhoea. Nat Rev Dis Primers..

[3] World Health Organization. Report on global sexually transmitted infection surveillance 2018. https://www.who.int/reproductivehealth/publications/stis-surveillance-2018/en/. Accessed 9 Oct 2020.

[4] Chan PA, Robinette A, Montgomery M, Almonte A, Cu-Uvin S, Lonks JR (2016). Extragenital infections caused by Chlamydia trachomatis and Neisseria gonorrhoeae: a review of the literature. Infect Dis Obstet Gynecol..

[5] Beck EJ, Mandalia S, Leonard K, Griffith RJ, Harris JR, Miller DL (1996). Case-control study of sexually transmitted diseases as cofactors for HIV-1 transmission. Int J STD AIDS..

[6] van Lier A, McDonald SA, Bouwknegt M, Kretzschmar ME, Havelaar AH, group EPI (2016). Disease burden of 32 infectious diseases in the Netherlands, 2007-2011. PLoS One..

[7] Satterwhite CL, Torrone E, Meites E, Dunne EF, Mahajan R, Ocfemia MC (2013). Sexually transmitted infections among US women and men: prevalence and incidence estimates, 2008. Sex Transm Dis..

[8] Leichliter JS, Dittus PJ, Copen CE, Aral SO (2020). Trends in factors indicating increased risk for STI among key subpopulations in the United States, 2002-2015. Sex Transm Infect..

[9] World Health Organization. Global Health Sector Strategy on Sexually Transmitted Infections 2016-2021. https://apps.who.int/iris/bitstream/handle/10665/246296/WHO-RHR-16.09-eng.pdf?sequence=1. Accessed 7 Oct 2020.

[10] Unemo M, Lahra MM, Cole M, Galarza P, Ndowa F, Martin I (2019). World Health Organization Global Gonococcal Antimicrobial Surveillance Program (WHO GASP): review of new data and evidence to inform international collaborative actions and research efforts. Sex Health..

[11] Spectrum. Glastonbury: Avenir Health 2019. https://www.avenirhealth.org/software-spectrum.php. Accessed 30 Nov 2020.

[12] Campbell M, McKenzie JE, Sowden A, Katikireddi SV, Brennan SE, Ellis S (2020). Synthesis without meta-analysis (SWiM) in systematic reviews: reporting guideline. BMJ..

[13] Behanzin L, Diabate S, Minani I, Lowndes CM, Boily MC, Labbe AC (2012). Decline in HIV prevalence among young men in the general population of Cotonou, Benin, 1998-2008. PLoS One..

[14] Paz-Soldan VA, Hoffman I, de Graft JJ, Bisika T, Kazembe PN, Feluzi H (2012). Sexually Transmitted Infection (STI) screening, case and contact treatment, and condom promotion resulting in STI reduction two years later in rural Malawi. Malawi Med J..

[15] Francis SC, Mthiyane TN, Baisley K, Mchunu SL, Ferguson JB, Smit T (2018). Prevalence of sexually transmitted infections among young people in South Africa: A nested survey in a health and demographic surveillance site. PLoS Med..

[16] de Lima YA, Turchi MD, Fonseca ZC, Garcia FL, de Brito E Cardoso FA, da Guarda Reis MN (2014). Sexually transmitted bacterial infections among young women in Central Western Brazil. Int J Infect Dis..

[17] Carcamo CP, Campos PE, Garcia PJ, Hughes JP, Garnett GP, Holmes KK (2012). Prevalences of sexually transmitted infections in young adults and female sex workers in Peru: a national population-based survey. Lancet Infect Dis..

[18] Sonnenberg P, Clifton S, Beddows S, Field N, Soldan K, Tanton C (2013). Prevalence, risk factors, and uptake of interventions for sexually transmitted infections in Britain: findings from the National Surveys of Sexual Attitudes and Lifestyles (Natsal). Lancet..

[19] Torrone EA, Johnson RE, Tian LH, Papp JR, Datta SD, Weinstock HS (2013). Prevalence of Neisseria gonorrhoeae among persons 14 to 39 years of age, United States, 1999 to 2008. Sex Transm Dis..

[20] Huai P, Li F, Li Z, Sun L, Fu X, Pan Q (2018). Prevalence, risk factors, and medical costs of Chlamydia trachomatis infections in Shandong Province, China: a population-based, cross-sectional study. BMC Infect Dis..

[21] Luo ZZ, Li W, Wu QH, Zhang L, Tian LS, Liu LL (2018). Population-based study of chlamydial and gonococcal infections among women in Shenzhen. China: Implications for programme planning. PLoS One.

[22] Corsenac P, Noel M, Rouchon B, Hoy D, Roth A (2015). Prevalence and sociodemographic risk factors of chlamydia, gonorrhoea and syphilis: a national multicentre STI survey in New Caledonia, 2012. BMJ Open..

[23] Mulu W, Yimer M, Zenebe Y, Abera B (2015). Common causes of vaginal infections and antibiotic susceptibility of aerobic bacterial isolates in women of reproductive age attending at Felegehiwot Referral Hospital. Ethiopia: a cross sectional study. BMC Womens Health.

[24] Tadesse E, Teshome M, Amsalu A, Shimelis T (2016). Genital Chlamydia trachomatis infection among women of reproductive age attending the gynecology clinic of Hawassa University Referral Hospital, Southern Ethiopia. PLoS One.

[25] Yirenya-Tawiah D, Annang TN, Apea-Kubi KA, Lomo G, Mensah D, Akyeh L (2014). Chlamydia Trachomatis and Neisseria Gonorrhoeae prevalence among women of reproductive age living in urogenital schistosomiasis endemic area in Ghana. BMC Res Notes..

[26] Jespers V, Crucitti T, Menten J, Verhelst R, Mwaura M, Mandaliya K (2014). Prevalence and correlates of bacterial vaginosis in different sub-populations of women in sub-Saharan Africa: a cross-sectional study. PLoS One..

[27] Kerubo E, Laserson KF, Otecko N, Odhiambo C, Mason L, Nyothach E (2016). Prevalence of reproductive tract infections and the predictive value of girls’symptom-based reporting: findings from a cross-sectional survey in rural western Kenya. Sex Transm Infect..

[28] Masese LN, Wanje G, Kabare E, Budambula V, Mutuku F, Omoni G (2017). Screening for sexually transmitted infections in adolescent girls and young women in Mombasa, Kenya: feasibility, prevalence, and correlates. Sex Transm Dis..

[29] Otieno FO, Ndivo R, Oswago S, Pals S, Chen R, Thomas T (2015). Correlates of prevalent sexually transmitted infections among participants screened for an HIV incidence cohort study in Kisumu, Kenya. Int J STD AIDS.

[30] Menendez C, Castellsague X, Renom M, Sacarlal J, Quinto L, Lloveras B (2010). Prevalence and risk factors of sexually transmitted infections and cervical neoplasia in women from a rural area of southern Mozambique. Infect Dis Obstet Gynecol..

[31] Kaida A, Dietrich JJ, Laher F, Beksinska M, Jaggernath M, Bardsley M (2018). A high burden of asymptomatic genital tract infections undermines the syndromic management approach among adolescents and young adults in South Africa: implications for HIV prevention efforts. BMC Infect Dis..

[32] Peters RPH, Dubbink JH, van der Eem L, Verweij SP, Bos MLA, Ouburg S (2014). Cross-sectional study of genital, rectal, and pharyngeal chlamydia and gonorrhea in women in rural South Africa. Sex Transm Dis..

[33] Rassjo EB, Mirembe F, Darj E (2011). Self-reported sexual behaviour among adolescent girls in Uganda: reliability of data debated. Afr Health Sci..

[34] Rutherford GW, Anglemyer A, Bagenda D, Muyonga M, Lindan CP, Barker JL (2014). University students and the risk of HIV and other sexually transmitted infections in Uganda: the Crane survey. Int J Adolesc Med Health..

[35] Offorjebe OA, Wynn A, Moshashane N, Joseph Davey D, Arena K, Ramogola-Masire D (2017). Partner notification and treatment for sexually transmitted infections among pregnant women in Gaborone, Botswana. Int J STD AIDS.

[36] Wynn A, Ramogola-Masire D, Gaolebale P, Moshashane N, Agatha Offorjebe O, Arena K (2016). Acceptability and feasibility of sexually transmitted infection testing and treatment among pregnant women in Gaborone, Botswana, 2015. Biomed Res Int..

[37] Masha SC, Wahome E, Vaneechoutte M, Cools P, Crucitti T, Sanders EJ (2017). High prevalence of curable sexually transmitted infections among pregnant women in a rural county hospital in Kilifi, Kenya. PLoS One.

[38] Warr AJ, Pintye J, Kinuthia J, Drake AL, Unger JA, McClelland RS (2019). Sexually transmitted infections during pregnancy and subsequent risk of stillbirth and infant mortality in Kenya: a prospective study. Sex Transm Infect..

[39] Abdelaziz ZA, Ibrahim ME, Bilal NE, Hamid ME (2014). Vaginal infections among pregnant women at Omdurman Maternity Hospital in Khartoum, Sudan. J Infect Dev Ctries.

[40] Abdelrahim NA, Ahmed HI, Fadl-Elmula IM, Bayoumi MA, Homeida MM (2017). Sexually transmitted infections other than HIV/AIDS among women of low socio-economic class attending antenatal clinics in Khartoum, Sudan. Int J STD AIDS.

[41] Chiduo M, Theilgaard ZP, Bakari V, Mtatifikolo F, Bygbjerg I, Flanholc L (2012). Prevalence of sexually transmitted infections among women attending antenatal clinics in Tanga, north eastern Tanzania. Int J STD AIDS..

[42] Hokororo A, Kihunrwa A, Hoekstra P, Kalluvya SE, Changalucha JM, Fitzgerald DW (2015). High prevalence of sexually transmitted infections in pregnant adolescent girls in Tanzania: a multi-community cross-sectional study. Sex Transm Infect..

[43] Chaponda EB, Chico RM, Bruce J, Michelo C, Vwalika B, Mharakurwa S (2016). Malarial infection and curable sexually transmitted and reproductive tract infections among pregnant women in a rural district of Zambia. Am J Trop Med Hyg..

[44] Piazzetta RC, de Carvalho NS, de Andrade RP, Piazzetta G, Piazzetta SR, Carneiro R (2011). Prevalence of Chlamydia trachomatis and Neisseria gonorrhoea infections in sexual actives young women at a southern Brazilian city. Rev Bras Ginecol Obstet..

[45] Pinto VM, Szwarcwald CL, Baroni C, Stringari LL, Inocencio LA, Miranda AE (2011). Chlamydia trachomatis prevalence and risk behaviors in parturient women aged 15 to 24 in Brazil. Sex Transm Dis..

[46] Rocha DA, Filho RA, Marino JM, dos Santos CM (2014). “Hidden” sexually transmitted infections among women in primary care health services, Amazonas, Brazil. Int J STD AIDS.

[47] Conejero C, Cannoni G, Merino PM, Bollmann J, Hidalgo C, Castro M (2013). Screening of Neisseria gonorrhoeae and Chlamydia trachomatis using techniques of self collected vaginal sample in young women. Rev Chil Infectol..

[48] Huneeus A, Schilling A, Fernandez MI (2018). Prevalence of Chlamydia trachomatis, Neisseria gonorrhoeae, and Trichomonas vaginalis infection in Chilean adolescents and young adults. J Pediatr Adolesc Gynecol..

[49] Paredes MC, Gomez YM, Torres AM, Fernandez M, Tovar MB (2015). Prevalence of infections by Chlamydia trachomatis and Neisseria gonorrhoeae among high school students in the Sabana Central area of Cundinamarca, Colombia. Biomedica.

[50] Jobe KA, Downey RF, Hammar D, Van Slyke L, Schmidt TA (2014). Epidemiology of sexually transmitted infections in rural southwestern Haiti: the Grand’nse Women’s Health Study. Am J Trop Med Hyg..

[51] Casillas-Vega N, Morfin-Otero R, Garcia S, Llaca-Diaz J, Rodriguez-Noriega E, Camacho-Ortiz A (2016). Sexually transmitted pathogens, coinfections and risk factors in patients attending obstetrics and gynecology clinics in Jalisco, Mexico. Salud Publica Mex.

[52] Silveira MF, Erbelding EJ, Ghanem KG, Johnson HL, Burke AE, Zenilman JM (2010). Risk of Chlamydia trachomatis infection during pregnancy: effectiveness of guidelines-based screening in identifying cases. Int J STD AIDS..

[53] Bristow CC, Mathelier P, Ocheretina O, Benoit D, Pape JW, Wynn A (2017). Chlamydia trachomatis, Neisseria gonorrhoeae, and Trichomonas vaginalis screening and treatment of pregnant women in Port-au-Prince, Haiti. Int J STD AIDS.

[54] Pourabbas B, Rezaei Z, Mardaneh J, Shahian M, Alborzi A (2018). Prevalence of Chlamydia trachomatis and Neisseria gonorrhoeae infections among pregnant women and eye colonization of their neonates at birth time, Shiraz, Southern Iran. BMC Infect Dis.

[55] Hassan SJ, Dunphy E, Navin E, Marron L, Fitzsimmons C, Loy A (2016). Screening for chlamydia is acceptable and feasible during cervical screening in general practice. Ir Med J..

[56] Matteelli A, Capelli M, Sulis G, Toninelli G, Carvalho AC, Pecorelli S (2016). Prevalence of Chlamydia trachomatis and Neisseria gonorrhoeae infection in adolescents in Northern Italy: an observational school-based study. BMC Public Health..

[57] Salfa MC, Suligoi B (2016). Prevalence of Chlamydia trachomatis, Trichomonas vaginalis and Neisseria gonorrhoeae based on data collected by a network of clinical microbiology laboratories, in Italy. Adv Exp Med Biol..

[58] Nolskog P, Backhaus E, Nasic S, Enroth H (2019). STI with Mycoplasma genitalium-more common than Chlamydia trachomatis in patients attending youth clinics in Sweden. Eur J Clin Microbiol Infect Dis..

[59] Sakem B, Michel R, Nydegger UE, Radjenovic D, Wydler M, Risch M (2011). Diagnostic relevance of simultaneous testing for Chlamydia trachomatis and Neisseria gonorrhoeae. Infection..

[60] Grech P, Marchant R, Samuel M (2017). Sexual health of women aged 40 and over attending an inner city integrated sexual health clinic. Int J STD AIDS..

[61] Peuchant O, Le Roy C, Desveaux C, Paris A, Asselineau J, Maldonado C (2015). Screening for Chlamydia trachomatis, Neisseria gonorrhoeae, and Mycoplasma genitalium should it be integrated into routine pregnancy care in French young pregnant women?. Diagn Microbiol Infect Dis..

[62] Borges-Costa J, Matos C, Pereira F (2012). Sexually transmitted infections in pregnant adolescents: prevalence and association with maternal and foetal morbidity. J Eur Acad Dermatol Venereol..

[63] Centers for Disease Control and Prevention (CDC). Sexually transmitted disease surveillance 2018: STDs in adolescents and young adults. https://www.cdc.gov/std/stats18/adolescents.htm. Accessed 9 Oct 2020.

[64] Jackson JA, McNair TS, Coleman JS (2015). Over-screening for chlamydia and gonorrhea among urban women age >/=25 years. Am J Obstet Gynecol.

[65] Newbern EC, Anschuetz GL, Eberhart MG, Salmon ME, Brady KA, De Los RA (2013). Adolescent sexually transmitted infections and risk for subsequent HIV. Am J Public Health..

[66] Nsuami MJ, Taylor SN (2012). Most adolescents who participate in school-based screenings for sexually transmitted infections do not perceive themselves at high risk of sexually transmitted infection. Int J STD AIDS..

[67] Akoh CC, Pressman EK, Cooper E, Queenan RA, Pillittere J, O’Brien KO (2017). Prevalence and risk factors for infections in a pregnant adolescent population. J Pediatr Adolesc Gynecol..

[68] Berggren EK, Patchen L (2011). Prevalence of Chlamydia trachomatis and Neisseria gonorrhoeae and repeat infection among pregnant urban adolescents. Sex Transm Dis..

[69] Blatt AJ, Lieberman JM, Hoover DR, Kaufman HW (2012). Chlamydial and gonococcal testing during pregnancy in the United States. Am J Obstet Gynecol.

[70] Waight MT, Rahman MM, Soto P, Tran T (2013). Sexually transmitted diseases during pregnancy in Louisiana, 2007-2009: high-risk populations and adverse newborn outcomes. J La State Med Soc..

[71] Krishnan A, Sabeena S, Bhat PV, Kamath V, Hindol M, Zadeh VR (2018). Detection of genital chlamydial and gonococcal infection using urine samples: A community-based study from India. J Infect Public Health..

[72] Asavapiriyanont S, Chaovarindr U, Kaoien S, Chotigeat U, Kovavisarach E (2016). Prevalence of sexually transmitted infection in teenage pregnancy in Rajavithi Hospital, Thailand. J Med Assoc Thail.

[73] Choe HS, Lee DS, Lee SJ, Lee CB, Lee WC, Cho YH (2012). Prevalence of sexually transmitted infections and sexual behavior of young adults and middle-aged people presenting to health examination centers in Korea. J Infect Chemother..

[74] Kim Y, Kim J, Lee KA (2014). Prevalence of sexually transmitted infections among healthy Korean women: implications of multiplex PCR pathogen detection on antibiotic therapy. J Infect Chemother..

[75] Vallely LM, Toliman P, Ryan C, Rai G, Wapling J, Gabuzzi J (2017). Performance of syndromic management for the detection and treatment of genital Chlamydia trachomatis, Neisseria gonorrhoeae and Trichomonas vaginalis among women attending antenatal, well woman and sexual health clinics in Papua New Guinea: a cross-sectional study. BMJ Open..

[76] Marks M, Kako H, Butcher R, Lauri B, Puiahi E, Pitakaka R (2015). Prevalence of sexually transmitted infections in female clinic attendees in Honiara, Solomon Islands. BMJ Open.

[77] Ekeroma AJ, Pandit L, Bartley C, Ikenasio-Thorpe B, Thompson JMD (2012). Screening for sexually transmitted infections in pregnancy at Middlemore Hospital, 2009. N Z Med J..

[78] Badman SG, Vallely LM, Toliman P, Kariwiga G, Lote B, Pomat W (2016). A novel point-of-care testing strategy for sexually transmitted infections among pregnant women in high-burden settings: results of a feasibility study in Papua New Guinea. BMC Infect Dis..

[79] Wangnapi RA, Soso S, Unger HW, Sawera C, Ome M, Umbers AJ (2015). Prevalence and risk factors for Chlamydia trachomatis, Neisseria gonorrhoeae and Trichomonas vaginalis infection in pregnant women in Papua New Guinea. Sex Transm Infect..

[80] Downey RF, Hammar D, Jobe KA, Schmidt TA, Slyke LV, Yassemi Y (2015). Epidemiology of sexually transmitted infections in rural Haitian men. Int J STD AIDS..

[81] Drinkard LN, Huxta RA, Halbritter A, Nguyen GT, Malebranche D (2017). The case for extragenital screening of Chlamydia trachomatis and Neisseria gonorrhoeae in the college health setting. Sex Transm Dis..

[82] Dave SS, Copas A, Richens J, White RG, Kosambiya JK, Desai VK (2012). HIV and STI prevalence and determinants among male migrant workers in India. PLoS One..

[83] Jatapai A, Sirivongrangson P, Lokpichat S, Chuenchitra T, Nelson KE, Rangsin R (2013). Prevalence and risk factors for Chlamydia trachomatis infection among young Thai men in 2008-2009. Sex Transm Dis..

[84] Zhang G, Wong M, Yi P, Xu J, Li B, Ding G (2010). HIV-1 and STIs prevalence and risk factors of miners in mining districts of Yunnan, China. J Acquir Immune Defic Syndr.

[85] European Centre for Disease Prevention and Control. Surveillance Atlas of Infectious Diseases. http://atlas.ecdc.europa.eu/public/index.aspx. Accessed 23 Nov 2020.

[86] European Centre for Disease Prevention and Control (ECDC). Gonorrhoea. Annual Epidemiological Report for 2017. https://www.ecdc.europa.eu/sites/portal/files/documents/gonorrhoea-annual-epidemiological-report-2017.pdf. Accessed 23 Nov 2020.

[87] Centers for Disease Control and Prevention (CDC). Sexually transmitted disease surveillance 2018: National profile - overview, Gonorrhea. https://www.cdc.gov/std/stats18/gonorrhea.htm. Accessed 9 Oct 2020.

[88] Government of Canada - Public Health Agency of Canada. Notifiable disease charts. https://diseases.canada.ca/notifiable/charts-list. Accessed 30 Nov 2020.

[89] World Health Organization. Report on global sexually transmitted infection surveillance 2015. http://apps.who.int/iris/bitstream/handle/10665/249553/9789241565301-eng.pdf;jsessionid=C5961C831C5BA711926D76292A64BAC2?sequence=1. Accessed 30 Nov 2020.

[90] Australasian Sexual Health Alliance. STI management guidelines for use in primary care. http://www.sti.guidelines.org.au/sexually-transmissible-infections/gonorrhoea. Accessed 30 Nov 2020.

[91] Ministry of Health Singapore. Blood-borne and sexually transmitted diseases. https://www.moh.gov.sg/docs/librariesprovider5/resources-statistics/reports/blood-borne-and-sexually-transmitted-diseases.pdf. Accessed 30 Nov 2020.

[92] Gonorrhoea writing group on behalf of the New Zealand Sexual Health Society. New Zealand guideline for the management of gonorrhoea, 2014, and response to the threat of antimicrobial resistance. https://nzshs.org/docman/guidelines/best-practice-guidelines/142-new-zealand-guideline-for-the-management-of-gonorrhoea-2014-and-response-to-the-threat-of-antimicrobial-resistance/file. Accessed 30 Nov 2020.

[93] National Institute of Infectious Diseases. NESID Annual Surveillance Data Sentinel-Reporting Diseaes 2015-3. https://www.niid.go.jp/niid/en/survei/2085-idwr/ydata/6552-report-eb2015-3.html. Accessed 30 Nov 2020.

[94] Korea Disease Control and Prevention Agency. Sexually Transmitted Infections (STIs) surveillance in the Republic of Korea, 2014-2018 - Public Health Weekly Report. http://www.cdc.go.kr/board/board.es?mid=a30501000000&bid=0031&list_no=366797&act=view. Accessed 17 Mar 2021.

[95] Tafuma TA, Merrigan MB, Okui LA, Lebelonyane R, Bolebantswe J, Mine M (2014). HIV/sexually transmitted infection prevalence and sexual behavior of men who have sex with men in 3 districts of Botswana: results from the 2012 biobehavioral survey. Sex Transm Dis..

[96] Sanders EJ, Wahome E, Okuku HS, Thiong’o AN, Smith AD, Duncan S (2014). Evaluation of WHO screening algorithm for the presumptive treatment of asymptomatic rectal gonorrhoea and chlamydia infections in at-risk MSM in Kenya. Sex Transm Infect..

[97] Sanders EJ, Okuku HS, Smith AD, Mwangome M, Wahome E, Fegan G (2013). High HIV-1 incidence, correlates of HIV-1 acquisition, and high viral loads following seroconversion among MSM. AIDS..

[98] Keshinro B, Crowell TA, Nowak RG, Adebajo S, Peel S, Gaydos CA (2016). High prevalence of HIV, chlamydia and gonorrhoea among men who have sex with men and transgender women attending trusted community centres in Abuja and Lagos, Nigeria. J Int AIDS Soc.

[99] Wade AS, Larmarange J, Diop AK, Diop O, Gueye K, Marra A (2010). Reduction in risk-taking behaviors among MSM in Senegal between 2004 and 2007 and prevalence of HIV and other STIs. ELIHoS Project, ANRS 12139. AIDS Care..

[100] Rebe K, Lewis D, Myer L, de Swardt G, Struthers H, Kamkuemah M, et al. A cross sectional analysis of gonococcal and chlamydial infections among men-who-have-sex-with-men in Cape Town, South Africa. PLoS One. 2015;10:e0138315. 10.1371/journal.pone.0138315 PMC4587970 26418464

[101] Ross MW, Nyoni J, Ahaneku HO, Mbwambo J, McClelland RS, McCurdy SA (2014). High HIV seroprevalence, rectal STIs and risky sexual behaviour in men who have sex with men in Dar es Salaam and Tanga, Tanzania. BMJ Open.

[102] Kim EJ, Hladik W, Barker J, Lubwama G, Sendagala S, Ssenkusu JM (2016). Sexually transmitted infections associated with alcohol use and HIV infection among men who have sex with men in Kampala, Uganda. Sex Transm Infect.

[103] Cunha CB, Friedman RK, de Boni RB, Gaydos C, Guimaraes MRC, Siqueira BH (2015). Chlamydia trachomatis, Neisseria gonorrhoeae and syphilis among men who have sex with men in Brazil. BMC Public Health.

[104] Creswell J, Guardado ME, Lee J, Nieto AI, Kim AA, Monterroso E (2012). HIV and STI control in El Salvador: results from an integrated behavioural survey among men who have sex with men. Sex Transm Infect..

[105] Figueroa JP, Weir SS, Jones-Cooper C, Byfield L, Hobbs MM, McKnight I (2013). High HIV prevalence among men who have sex with men in Jamaica is associated with social vulnerability and other sexually transmitted infections. West Indian Med J..

[106] Allan-Blitz LT, Leon SR, Bristow CC, Konda KA, Vargas SK, Flores JA (2017). High prevalence of extra-genital chlamydial or gonococcal infections among men who have sex with men and transgender women in Lima, Peru. Int J STD AIDS.

[107] Castillo R, Konda KA, Leon SR, Silva-Santisteban A, Salazar X, Klausner JD (2015). HIV and sexually transmitted infection incidence and associated risk factors among high-risk MSM and male-to-female transgender women in Lima, Peru. J Acquir Immune Defic Syndr.

[108] Kojima N, Park H, Konda KA, Joseph Davey DL, Bristow CC, Brown B (2017). The PICASSO Cohort: baseline characteristics of a cohort of men who have sex with men and male-to-female transgender women at high risk for syphilis infection in Lima, Peru. BMC Infect Dis.

[109] Leon SR, Segura ER, Konda KA, Flores JA, Silva-Santisteban A, Galea JT (2016). High prevalence of Chlamydia trachomatis and Neisseria gonorrhoeae infections in anal and pharyngeal sites among a community-based sample of men who have sex with men and transgender women in Lima, Peru. BMJ Open.

[110] Perez-Brumer AG, Konda KA, Salvatierra HJ, Segura ER, Hall ER, Montano SM (2013). Prevalence of HIV, STIs, and risk behaviors in a cross-sectional community- and clinic-based sample of men who have sex with men (MSM) in Lima, Peru. PLoS One.

[111] Dudareva-Vizule S, Haar K, Sailer A, Wisplinghoff H, Wisplinghoff F, Marcus U (2014). Prevalence of pharyngeal and rectal Chlamydia trachomatis and Neisseria gonorrhoeae infections among men who have sex with men in Germany. Sex Transm Infect..

[112] Marcus U, Ort J, Grenz M, Eckstein K, Wirtz K, Wille A (2015). Risk factors for HIV and STI diagnosis in a community-based HIV/STI testing and counselling site for men having sex with men (MSM) in a large German city in 2011-2012. BMC Infect Dis..

[113] Foschi C, Gaspari V, Sgubbi P, Salvo M, D’Antuono A, Marangoni A (2018). Sexually transmitted rectal infections in a cohort of ‘men having sex with men’. J Med Microbiol..

[114] Heiligenberg M, Wermeling PR, van Rooijen MS, Urbanus AT, Speksnijder AGCL, Heijman T (2012). Recreational drug use during sex and sexually transmitted infections among clients of a city sexually transmitted infections clinic in Amsterdam, the Netherlands. Sex Transm Dis..

[115] van Liere GAFS, Hoebe CJPA, Dukers-Muijrers NHTM (2014). Evaluation of the anatomical site distribution of chlamydia and gonorrhoea in men who have sex with men and in high-risk women by routine testing: cross-sectional study revealing missed opportunities for treatment strategies. Sex Transm Infect..

[116] van Liere GAFS, van Rooijen MS, Hoebe CJPA, Heijman T, de Vries HJ, Dukers-Muijrers NHTM (2015). Prevalence of and factors associated with rectal-only chlamydia and gonorrhoea in women and in men who have sex with men. PLoS One..

[117] Soni S, Alexander S, Verlander N, Saunders P, Richardson D, Fisher M (2010). The prevalence of urethral and rectal Mycoplasma genitalium and its associations in men who have sex with men attending a genitourinary medicine clinic. Sex Transm Infect..

[118] Gratrix J, Singh AE, Bergman J, Egan C, McGinnis J, Drews SJ (2014). Prevalence and characteristics of rectal chlamydia and gonorrhea cases among men who have sex with men after the introduction of nucleic acid amplification test screening at 2 Canadian sexually transmitted infection clinics. Sex Transm Dis..

[119] Remis RS, Liu J, Loutfy MR, Tharao W, Rebbapragada A, Huibner S (2016). Prevalence of sexually transmitted viral and bacterial infections in HIV-positive and HIV-negative men who have sex with men in Toronto. PLoS One..

[120] Anschuetz GL, Paulukonis E, Powers R, Asbel LE (2016). Extragenital screening in men who have sex with men diagnoses more chlamydia and gonorrhea cases than urine testing alone. Sex Transm Dis..

[121] Hassan A, Blumenthal JS, Dube MP, Ellorin E, Corado K, Moore DJ (2018). Effect of rectal douching/enema on rectal gonorrhoea and chlamydia among a cohort of men who have sex with men on HIV pre-exposure prophylaxis. Sex Transm Infect..

[122] Mayer KH, Ducharme R, Zaller ND, Chan PA, Case P, Abbott D (2012). Unprotected sex, underestimated risk, undiagnosed HIV and sexually transmitted diseases among men who have sex with men accessing testing services in a New England bathhouse. J Acquir Immune Defic Syndr..

[123] Montano MA, Dombrowski JC, Dasgupta S, Golden MR, Duerr A, Manhart LE (2019). Changes in sexual behavior and STI diagnoses among MSM initiating PrEP in a clinic setting. AIDS Behav..

[124] Mustanski B, Feinstein BA, Madkins K, Sullivan P, Swann G (2017). Prevalence and risk factors for rectal and urethral sexually transmitted infections from self-collected samples among young men who have sex with men participating in the Keep it up! 2.0 randomized controlled trial. Sex Transm Dis..

[125] Patton ME, Kidd S, Llata E, Stenger M, Braxton J, Asbel L (2014). Extragenital gonorrhea and chlamydia testing and infection among men who have sex with men--STD Surveillance Network, United States, 2010-2012. Clin Infect Dis..

[126] Sexton ME, Baker JJ, Nakagawa K, Li Y, Perkins R, Slack RS (2013). How reliable is self-testing for gonorrhea and chlamydia among men who have sex with men?. J Fam Pract..

[127] Taylor MM, Newman DR, Gonzalez J, Skinner J, Khurana R, Mickey T (2015). HIV status and viral loads among men testing positive for rectal gonorrhoea and chlamydia, Maricopa County, Arizona, USA, 2011-2013. HIV Med..

[128] Aggarwal P, Bhattar S, Sahani SK, Bhalla P, Garg VK (2016). Sexually transmitted infections and HIV in self reporting men who have sex with men: A two-year study from India. J Infect Public Health..

[129] Hananta IP, van Dam AP, Bruisten SM, van der Loeff MFS, Soebono H, de Vries HJ (2016). Gonorrhea in Indonesia: high prevalence of asymptomatic urogenital gonorrhea but no circulating extended spectrum cephalosporins-resistant Neisseria gonorrhoeae strains in Jakarta, Yogyakarta, and Denpasar, Indonesia. Sex Transm Dis.

[130] Morineau G, Nugrahini N, Riono P, Nurhayati GP, Mustikawati DE (2011). Sexual risk taking, STI and HIV prevalence among men who have sex with men in six Indonesian cities. AIDS Behav..

[131] Pattanasin S, Dunne EF, Wasinrapee P, Tongtoyai J, Chonwattana W, Sriporn A (2018). Screening for Chlamydia trachomatis and Neisseria gonorrhoeae infection among asymptomatic men who have sex with men in Bangkok, Thailand. Int J STD AIDS.

[132] Tongtoyai J, Todd CS, Chonwattana W, Pattanasin S, Chaikummao S, Varangrat A (2015). Prevalence and correlates of Chlamydia trachomatis and Neisseria gonorrhoeae by anatomic site among urban Thai men who have sex with men. Sex Transm Dis..

[133] Chow EPF, Tomnay J, Fehler G, Whiley D, Read TR, Denham I (2015). Substantial increases in chlamydia and gonorrhea positivity unexplained by changes in individual-level sexual behaviors among men who have sex with men in an Australian sexual health service from 2007 to 2013. Sex Transm Dis..

[134] Nash JL, Hocking JS, Read TR, Chen MY, Bradshaw CS, Forcey DS (2014). Contribution of sexual practices (other than anal sex) to bacterial sexually transmitted infection transmission in men who have sex with men: a cross-sectional analysis using electronic health records. Sex Transm Infect..

[135] Ong JJ, Fethers K, Howden BP, Fairley CK, Chow EPF, Williamson DA (2017). Asymptomatic and symptomatic urethral gonorrhoea in men who have sex with men attending a sexual health service. Clin Microbiol Infect..

[136] Ryder N, Lockart IG, Bourne C (2010). Is screening asymptomatic men who have sex with men for urethral gonorrhoea worthwhile?. Sex Health..

[137] Vodstrcil LA, Fairley CK, Fehler G, Leslie D, Walker J, Bradshaw CS (2011). Trends in chlamydia and gonorrhea positivity among heterosexual men and men who have sex with men attending a large urban sexual health service in Australia, 2002-2009. BMC Infect Dis..

[138] Yang TZT, Chen MY, Read TRH, Needleman R, Bradshaw CS, Fortune R (2018). Sampling technique and detection rates of oropharyngeal and anorectal gonorrhoea using nucleic acid amplification tests in men who have sex with men. Sex Transm Infect..

[139] Chen X, Fu G, Xu X, Hu H, Zuo H, Liu X, et al. Study on gonococcal and chlamydial infections among men who have sex with men in Nanjin. Acta Universitatis Medicinalis Anhui. 2011:569–72.

[140] Fu GF, Jiang N, Hu HY, Mahapatra T, Yin YP, Mahapatra S (2015). The epidemic of HIV, syphilis, chlamydia and gonorrhea and the correlates of sexual transmitted infections among men who have sex with men in Jiangsu, China, 2009. PLoS One..

[141] Guo Y, Wang D, Zhou J, Chen S, Wang J, Zhen S (2014). Effects of education level of men who have sex with men on their high risk sexual behaviors and the infection of HIV and syphilis. Zhonghua Yu Fang Yi Xue Za Zhi..

[142] Huan XP, Yin YP, Fu GF, Jiang N, Zhang QQ, Zhang XN (2011). Analysis on sexually transmitted diseases and the related risk factors among men who have sex with men in Jiangsu province. Zhonghua Yu Fang Yi Xue Za Zhi..

[143] Liu YJ, Jiang SL, Hu Y, Song L, Yu M, Li SM (2011). Characteristics of sexual behaviors and infection status of AIDS and other sexually transmitted diseases among men who have sex with men in 2009 in Beijing. Zhonghua Yu Fang Yi Xue Za Zhi..

[144] Yang LG, Zhang XH, Zhao PZ, Chen ZY, Ke WJ, Ren XQ (2018). Gonorrhea and chlamydia prevalence in different anatomical sites among men who have sex with men: a cross-sectional study in Guangzhou. China. BMC Infect Dis.

[145] Zhang X, Jia M, Chen M, Luo H, Chen H, Luo W (2017). Prevalence and the associated risk factors of HIV, STIs and HBV among men who have sex with men in Kunming, China. Int J STD AIDS.

[146] Jung M, Lee J, Kwon DS, Park BJ (2012). Comparison of sexual risky factors of men who have sex with men and sex-buying men as groups vulnerable to sexually transmitted diseases. J Prev Med Public Health..

[147] Pham QD, Nguyen TV, Hoang CQ, Cao V, Khuu NV, Phan HT (2012). Prevalence of HIV/STIs and associated factors among men who have sex with men in An Giang, Vietnam. Sex Transm Dis.

[148] Beymer MR, Weiss RE, Bolan RK, Rudy ET, Bourque LB, Rodriguez JP (2014). Sex on demand: geosocial networking phone apps and risk of sexually transmitted infections among a cross-sectional sample of men who have sex with men in Los Angeles County. Sex Transm Infect..

[149] Cheung KT, Fairley CK, Read TRH, Denham I, Fehler G, Bradshaw CS (2016). HIV incidence and predictors of incident HIV among men who have sex with men attending a sexual health clinic in Melbourne, Australia. PLoS One.

[150] Pinsky L, Chiarilli DB, Klausner JD, Kull RM, O’Keefe R, Heffer C (2012). Rates of asymptomatic nonurethral gonorrhea and chlamydia in a population of university men who have sex with men. J Am Coll Heal..

[151] Behanzin L, Diabate S, Minani I, Boily MC, Labbe AC, Ahoussinou C (2013). Decline in the prevalence of HIV and sexually transmitted infections among female sex workers in Benin over 15 years of targeted interventions. J Acquir Immune Defic Syndr..

[152] Merrigan MB, Tafuma TA, Okui LA, Lebelonyane R, Bolebantswe JM, Makhaola K (2015). HIV prevalence and risk behaviors among female sex workers in Botswana: results from the 2012 HIV/STI bio-behavioral study. AIDS Behav..

[153] Vuylsteke B, Semde G, Sika L, Crucitti T, Ettiegne Traore V, Buve A (2012). HIV and STI prevalence among female sex workers in Cote d’Ivoire: why targeted prevention programs should be continued and strengthened. PLoS One..

[154] Tadele A, Hussen S, Shimelis T (2019). Prevalence and associated factors of Chlamydia trachomatis and Neisseria gonorrhoeae among female commercial sex workers in Hawassa City, Southern Ethiopia. BMC Infect Dis.

[155] Aho J, Koushik A, Coutlee F, Diakite SL, Rashed S (2014). Prevalence of HIV, human papillomavirus type 16 and herpes simplex virus type 2 among female sex workers in Guinea and associated factors. Int J STD AIDS..

[156] Izulla P, McKinnon LR, Munyao J, Karanja S, Koima W, Parmeres J (2013). HIV postexposure prophylaxis in an urban population of female sex workers in Nairobi, Kenya. J Acquir Immune Defic Syndr.

[157] Braunstein SL, Ingabire CM, Kestelyn E, Uwizera AU, Mwamarangwe L, Ntirushwa J (2011). High human immunodeficiency virus incidence in a cohort of Rwandan female sex workers. Sex Transm Dis..

[158] Vandepitte J, Muller E, Bukenya J, Nakubulwa S, Kyakuwa N, Buve A (2012). Prevalence and correlates of Mycoplasma genitalium infection among female sex workers in Kampala, Uganda. J Infect Dis.

[159] Sabido M, Lahuerta M, Montoliu A, Gonzalez V, Hernandez G, Giardina F (2011). Human immunodeficiency virus, sexually transmitted infections, and risk behaviors among clients of sex workers in Guatemala: are they a bridge in human immunodeficiency virus transmission?. Sex Transm Dis..

[160] Tinajeros F, Miller WM, Castro L, Artiles N, Flores F, Evans JL (2012). Declining sexually transmitted infections among female sex workers: the results of an HIV and sexually transmitted infection prevention strategy in Honduras, 2006-08. Int J STD AIDS..

[161] Bazzi AR, Rangel G, Martinez G, Ulibarri MD, Syvertsen JL, Bazzi SA (2015). Incidence and predictors of HIV and sexually transmitted infections among female sex workers and their intimate male partners in northern Mexico: a longitudinal, multilevel study. Am J Epidemiol..

[162] Kazerooni PA, Motazedian N, Motamedifar M, Sayadi M, Sabet M, Lari MA (2014). The prevalence of human immunodeficiency virus and sexually transmitted infections among female sex workers in Shiraz, South of Iran: by respondent-driven sampling. Int J STD AIDS..

[163] Nasirian M, Kianersi S, Hoseini SG, Kassaian N, Yaran M, Shoaei P (2017). Prevalence of sexually transmitted infections and their risk factors among female sex workers in Isfahan, Iran: a cross-sectional study. J Int Assoc Provid AIDS Care..

[164] Khan MS, Unemo M, Zaman S, Lundborg CS (2011). HIV, STI prevalence and risk behaviours among women selling sex in Lahore, Pakistan. BMC Infect Dis.

[165] Znazen A, Frikha-Gargouri O, Berrajah L, Bellalouna S, Hakim H, Gueddana N (2010). Sexually transmitted infections among female sex workers in Tunisia: high prevalence of Chlamydia trachomatis. Sex Transm Infect..

[166] Mc Grath-Lone L, Marsh K, Hughes G, Ward H (2014). The sexual health of female sex workers compared with other women in England: analysis of cross-sectional data from genitourinary medicine clinics. Sex Transm Infect..

[167] Haseen F, Chawdhury FA, Hossain ME, Huq M, Bhuiyan MU, Imam H (2012). Sexually transmitted infections and sexual behaviour among youth clients of hotel-based female sex workers in Dhaka, Bangladesh. Int J STD AIDS.

[168] Khanam R, Reza M, Ahmed D, Rahman M, Alam MS, Sultana S (2017). Sexually transmitted infections and associated risk factors among street-based and residence-based female sex workers in Dhaka, Bangladesh. Sex Transm Dis.

[169] Das A, Pathni AK, Narayanan P, George B, Morineau G, Saidel T (2013). High rates of reinfection and incidence of bacterial sexually transmitted infections in a cohort of female sex workers from two Indian cities: need for different STI control strategies?. Sex Transm Infect..

[170] Hemalatha R, Kumar RH, Venkaiah K, Srinivasan K, Brahmam GN (2011). Prevalence of & knowledge, attitude & practices towards HIV & sexually transmitted infections (STIs) among female sex workers (FSWs) in Andhra Pradesh. Indian J Med Res..

[171] Bollen LJ, Anartati AS, Morineau G, Sulami S, Prabawanti C, Silfanus FJ (2010). Addressing the high prevalence of gonorrhoea and chlamydia among female sex workers in Indonesia: results of an enhanced, comprehensive intervention. Sex Transm Infect..

[172] Majid N, Bollen L, Morineau G, Daily SF, Mustikawati DE, Agus N (2010). Syphilis among female sex workers in Indonesia: need and opportunity for intervention. Sex Transm Infect..

[173] Mawu FO, Davies SC, McKechnie M, Sedyaningsih ER, Widihastuti A, Hillman RJ (2011). Sexually transmissible infections among female sex workers in Manado, Indonesia, using a multiplex polymerase chain reaction-based reverse line blot assay. Sex Health..

[174] Silitonga N, Davies SC, Kaldor J, Wignall S, Okoseray M (2011). Prevalence over time and risk factors for sexually transmissible infections among newly-arrived female sex workers in Timika, Indonesia. Sex Health.

[175] Tanudyaya FK, Rahardjo E, Bollen LJ, Madjid N, Daili SF, Priohutomo S (2010). Prevalence of sexually transmitted infections and sexual risk behavior among female sex workers in nine provinces in Indonesia, 2005. Southeast Asian J Trop Med Public Health..

[176] Couture MC, Sansothy N, Sapphon V, Phal S, Sichan K, Stein E (2011). Young women engaged in sex work in Phnom Penh, Cambodia, have high incidence of HIV and sexually transmitted infections, and amphetamine-type stimulant use: new challenges to HIV prevention and risk. Sex Transm Dis..

[177] Chen XS, Yin YP, Liang GJ, Wang QQ, Jiang N, Liu Q (2013). The prevalences of Neisseria gonorrhoeae and Chlamydia trachomatis infections among female sex workers in China. BMC Public Health..

[178] Guo Y, Xu X, Fu G, Huan X, Jiang N, Yin Y (2017). Risk behaviours and prevalences of HIV and sexually transmitted infections among female sex workers in various venues in Changzhou, China. Int J STD AIDS.

[179] Jin X, Chan S, Ding G, Wang H, Xu J, Wang G (2011). Prevalence and risk behaviours for Chlamydia trachomatis and Neisseria gonorrhoeae infection among female sex workers in an HIV/AIDS high-risk area. Int J STD AIDS..

[180] Luo L, Li X, Zhang LL (2015). Neisseria gonorrhoeae prevalence, incidence and associated risk factors among female sex workers in a high HIV-prevalence area of China. Int J Infect Dis..

[181] Remis RS, Kang L, Calzavara L, Pan Q, Liu J, Myers T (2015). Prevalence and correlates of HIV infection and sexually transmitted infections in female sex workers (FSWs) in Shanghai, China. Epidemiol Infect.

[182] Tang W, Pan J, Jiang N, Hu HY, Mahapatra T, Yin YP (2014). Correlates of chlamydia and gonorrhea infection among female sex workers: the untold story of Jiangsu, China. PLoS One.

[183] Wong WC, Yim YL, Lynn H (2011). Sexually transmitted infections among female sex workers in Hong Kong: the role of migration status. J Travel Med..

[184] Wong HT, Lee KC, Chan DP (2015). Community-based sexually transmitted infection screening and increased detection of pharyngeal and urogenital Chlamydia trachomatis and Neisseria gonorrhoeae infections in female sex workers in Hong Kong. Sex Transm Dis..

[185] Zhu BY, Bu J, Huang PY, Zhou ZG, Yin YP, Chen XS (2012). Epidemiology of sexually transmitted infections, HIV, and related high-risk behaviors among female sex workers in Guangxi Autonomous Region, China. Jpn J Infect Dis.

[186] Vuylsteke B, Semde G, Sika L, Crucitti T, Ettiegne Traore V, Buve A (2012). High prevalence of HIV and sexually transmitted infections among male sex workers in Abidjan, Cote d’Ivoire: need for services tailored to their needs. Sex Transm Infect..

[187] Galarraga O, Sosa-Rubi SG, Gonzalez A, Badial-Hernandez F, Conde-Glez CJ, Juarez-Figueroa L (2014). The disproportionate burden of HIV and STIs among male sex workers in Mexico City and the rationale for economic incentives to reduce risks. J Int AIDS Soc..

[188] Goldsamt LA, Clatts MC, Giang LM, Le BQ, Colby DJ, Yu G (2018). HIV and other STIs in male sex workers: Findings from a sexual health promotion intervention in Vietnam. Int J STD AIDS..

[189] Chow EP, Tucker JD, Wong FY, Nehl EJ, Wang Y, Zhuang X (2014). Disparities and risks of sexually transmissible infections among men who have sex with men in China: a meta-analysis and data synthesis. PLoS One..

[190] Institute of Health Metrics and Evaluation (IHME). Findings from the Global Burden of Disease Study 2017. http://www.healthdata.org/policy-report/findings-global-burden-disease-study-2017. Accessed 30 Nov 2020.

[191] World Health Organization. Standard protocol to assess prevalence of gonorrhoea and chlamydia among pregnant women in antenatal care clinics. https://apps.who.int/iris/handle/10665/275846. Accessed 30 Nov 2020.

[192] Vandenbroucke JP, von Elm E, Altman DG, Gøtzsche PC, Mulrow CD, Pocock SJ (2007). Strengthening the Reporting of Observational Studies in Epidemiology (STROBE): explanation and elaboration. PLoS Med..

