# Supplementary material for: Gonorrhoea: a systematic review of prevalence reporting globally
Source: BMC Infect Dis. 2021 Nov 11;21:1152. doi: 10.1186/s12879-021-06381-4 (PMC8582208; doi:10.1186/s12879-021-06381-4)
Supplement: Supplementary file 2 — Additional file 2. Literature search strategy. [file 12879_2021_6381_MOESM2_ESM.pdf]

Additional file 2. Literature search strategy.

Search Strategy	Terms	PubMed Search	Number of records retrieved
MeSH terms	"Sexually Transmitted Diseases/epidemiology" [Majr] OR<br>"Sexually Transmitted Diseases, Bacterial/epidemiology" [Majr] OR<br>"Gonorrhea/diagnosis"[MeSH] OR<br>"Gonorrhea/epidemiology"[MeSH] OR<br>"Neisseria gonorrhoeae/diagnosis"[MeSH] OR<br>"Neisseria gonorrhoeae/epidemiology" [MeSH]	("Sexually Transmitted Diseases/epidemiology"[Majr] OR "Sexually Transmitted Diseases, Bacterial/epidemiology"[Majr] OR "Gonorrhea/diagnosis"[MeSH] OR "Gonorrhea/epidemiology"[MeSH] OR "Neisseria gonorrhoeae/diagnosis"[MeSH] OR "Neisseria gonorrhoeae/epidemiology"[MeSH])	43 713
Keywords	Gonorrh*[tiab] OR<br>gonococc* [tiab]	(gonorr* [tiab] OR gonococc* [tiab])	22 541
MeSH terms and Keywords combined with AND		(((((gonorr* [tiab] OR gonococc* [tiab])))) AND ((("Sexually Transmitted Diseases/epidemiology"[Majr] OR "Sexually Transmitted Diseases, Bacterial/epidemiology"[Majr] OR "Gonorrhea/diagnosis"[MeSH] OR "Gonorrhea/epidemiology"[MeSH] OR "Neisseria gonorrhoeae/diagnosis"[MeSH] OR "Neisseria gonorrhoeae/epidemiology"[MeSH])))	6520
Mesh terms AND Keywords, with limitation of publication date (1 January 2010 to 11 April 2019)		(((((gonorr* [tiab] OR gonococc* [tiab])))) AND ((("Sexually Transmitted Diseases/epidemiology"[Majr] OR "Sexually Transmitted Diseases, Bacterial/epidemiology"[Majr] OR "Gonorrhea/diagnosis"[MeSH] OR "Gonorrhea/epidemiology"[MeSH] OR "Neisseria gonorrhoeae/diagnosis"[MeSH] OR "Neisseria gonorrhoeae/epidemiology"[MeSH]))) AND ("2010/01/01"[Date - Publication] : "2019/04/11"[Date - Publication]))	**2015**

MESH=Medical subject headings. NA=not applicable.
