# Supplementary material for: Gonorrhoea: a systematic review of prevalence reporting globally
Source: BMC Infect Dis. 2021 Nov 11;21:1152. doi: 10.1186/s12879-021-06381-4 (PMC8582208; doi:10.1186/s12879-021-06381-4)
Supplement: Supplementary file 3 — Additional file3. Inclusion and exclusion criteria. [file 12879_2021_6381_MOESM3_ESM.pdf]

Additional file 3. Inclusion and exclusion criteria.

Category	Inclusion criteria	Exclusion criteria
General	Published between 1 January 2010 and 11 April 2019.	Published outside specified time limits.
Publications	Primary publications in peer-reviewed journals or original data published in international, regional or national surveillance reports or guidelines (WHO, ECDC, Japanese Infectious Disease Surveillance Centre, Ministry of Health Singapore, Republic of Korea CDC, New Zealand Public Health Surveillance, Australia Department of Health, Public Health Agency of Canada, USA CDC).	Secondary publications or literature reviews. Reference lists of literature reviews were scanned and if meeting the inclusion criteria, the primary publication was included.
Language	English language abstract. If abstract was relevant, full text articles in all languages were eligible.	Non-English language abstract.
Study outcome	Prevalence of gonorrhoea defined as the proportion of positive tests per number of persons tested at the study level.	Incidence rates reported per person-time. Where screening is advised for subgroups of the general population, but it was unclear whether screening was applied opportunistically or universally, these reports were excluded, with the exception of MSM and sex workers.
Data source	Studies reporting data that might reasonably be generalized to the population addressed in the study hypothesis, e.g. reporting from cross-sectional/cohort data from empirical studies reporting laboratory-confirmed infections.	Data published in more than one paper or duplicate of an existing entry; data from a non-specified/non-validated data source; secondary analyses on existing (previously collected) data aimed to determine risk factors associated with STIs such as gonorrhoea; modelling studies.
Sample size	Sample size >100.	Sample size <100. Exceptions were made for MSM and sex worker populations in countries where data was otherwise limited or absent.
Study population	Patients with laboratory-confirmed gonorrhoea in one or more of the three population subgroups: general population samples that might reasonably be representative of the source population (men and women, according to WHO recommendations. Examples include pregnant women, women attending family planning clinics, military recruits, and men undergoing employment physicals), MSM or sex workers.	For general population: Subset of the population or subgroup that might reasonably reflect a different level of risk than in the general population: children or minors; studies including only symptomatic patients, or conducted in some clinic settings or in women attending gynaecology or sexual health clinics with STI symptoms; 'niche' subpopulations not typically representative of the target populations, e.g. homeless, refugees, military in residential settings, minority or remote ethnic groups.<br>For MSM and sex workers: Not population of interest, e.g. bisexual or transgender population, or only HIV-positive population.
Study Setting	Population-based studies including community recruitment (household surveys, electoral register), general practices, antenatal clinics, family planning clinics, non-'niche' populations, and for men only: non-residential occupational groups, e.g. studies of military were only included if findings were likely representative of the wider male population, including random sampling of conscripts versus soldiers in barracks/shared residential setting.	For general population: Settings where prevalence is likely different than in the general population, e.g. STI clinics, prisons, infertility clinics, emergency department attendees.
	For MSM and sex workers: These groups are defined according to their sexual behaviour and thus, studies included those conducted in STI clinics but excluded those with a symptomatic presentation.	For MSM and sex workers: Settings where prevalence is likely different than in the target population, e.g. prisons, emergency department attendees.
Information in title and abstract	The title and abstract provide enough information for a decision.	The title and abstract cannot provide enough information for a decision.
Infection	Laboratory-confirmed infection (including self-swabbing) using testing including culture, and NAAT applied to recommended clinical specimens (urine, urogenital swabs, rectal and pharyngeal swabs).	Self-reported (unconfirmed) infection; composite endpoints: e.g. gonorrhoea and/or chlamydia, infection of the eye, neonatal gonorrhoea or anatomic sites not specified.

CDC=Centers for Disease Control and Prevention. ECDC=European Centre for Disease Prevention and Control. HIV=human immunodeficiency virus. MSM=men-who-have-sex-with-men. NAAT=nucleic acid amplification tests. STI=sexually transmitted infection. WHO=World Health Organization.
