# Supplementary material for: Gonorrhoea: a systematic review of prevalence reporting globally
Source: BMC Infect Dis. 2021 Nov 11;21:1152. doi: 10.1186/s12879-021-06381-4 (PMC8582208; doi:10.1186/s12879-021-06381-4)
Supplement: Supplementary file 4 — Additional file 4. Standardizations adopted for urogenital, rectal and pharyngeal laboratory tests. [file 12879_2021_6381_MOESM4_ESM.pdf]

Additional file 4. Standardizations adopted for urogenital, rectal and pharyngeal laboratory tests.

Standardization was conducted for urogenital infection using the same standardization factors used by the SPECTRUM tool for WHO since 2018 [1, 2]. The standardized prevalence (SP) estimate was obtained through adjustment of the study reported prevalence (RP) for the sensitivity and specificity of the diagnostic test (NAAT or culture), the clinical specimen (urine or urogenital fluid) and the geographic location (urban or rural). The following formula was applied:

$$SP = \frac{(RP + Specificity - 1)}{(Sensitivity + Specificity - 1)} \times geography\ adjustor$$

The sensitivities and specificities that we applied are summarized as follows:

Sex	Specimen	Test	Sensitivity, %	Specificity, %
Women	Genital fluid	NAAT	93.3	99.2
		Culture	75.7	100
	Urine	NAAT	91.6	100
Men	Genital fluid	NAAT	96.1	99.0
		Culture	87.6	100
	Urine	NAAT	80.9	99.9

NAAT=nucleic acid amplification test.

The geographic adjustor was applied for urban (0.947), and rural (1.052) areas. If national or unknown, no geographic adjustment was made.

There are currently no WHO-endorsed standardizations for sensitivities and specificities of laboratory tests for rectal or pharyngeal gonococcal infections. In a targeted review of the literature, we reviewed sensitivities and specificities of diagnostic tests detecting *Neisseria gonorrhoeae* in rectal and/or pharyngeal samples among different populations [3-11]. Particularly for the rectal samples, there was considerable diversity in the sensitivity and specificity depending on the symptom status of the participants included in the sample, different brands of NAAT, or whether the sample was self- or clinician-collected. Culture sensitivity also differed in different settings (notably for culture samples between men and women (not tabulated), results were similar [5]). Based on the outcome of the literature review, we applied conservative estimated standardizations for rectal samples as follows: NAAT sensitivity: 90%, NAAT specificity: 98%; culture sensitivity: 60%, culture specificity: 100%. We applied the same standardizations for pharyngeal samples because appropriate validation studies were few and most studies reported the use of Food and Drug Administration-approved NAATs. The high prevalence rates of pharyngeal gonorrhoea in the very few studies that did not report the diagnostic test for pharyngeal samples also suggest the use of adequate NAATs.

References Additional file 4

[1] Spectrum. Glastonbury: Avenir Health 2019. https://www.avenirhealth.org/software-spectrum.php. Accessed 30 November 2020.
[2] World Health Organization. Report on global sexually transmitted infection surveillance 2018. https://www.who.int/reproductivehealth/publications/stis-surveillance-2018/en/. Accessed 9 October 2020.
[3] Bachmann LH, Johnson RE, Cheng H, Markowitz L, Papp JR, Palella FJ, Jr., et al. Nucleic acid amplification tests for diagnosis of Neisseria gonorrhoeae and Chlamydia trachomatis rectal infections. J Clin Microbiol. 2010;48:1827–32.
[4] Cosentino LA, Danby CS, Rabe LK, Macio I, Meyn LA, Wiesenfeld HC, et al. Use of nucleic acid amplification testing for diagnosis of extragenital sexually transmitted infections. J Clin Microbiol. 2017;55:2801–7.
[5] Harryman L, Scofield S, Macleod J, Carrington D, Williams OM, Fernandes A, et al. Comparative performance of culture using swabs transported in Amies medium and the Aptima Combo 2 nucleic acid amplification test in detection of Neisseria gonorrhoeae from genital and extra-genital sites: a retrospective study. Sex Transm Infect. 2012;88:27–31.
[6] Moncada J, Schachter J, Liska S, Shayevich C, Klausner JD. Evaluation of self-collected glans and rectal swabs from men who have sex with men for detection of Chlamydia trachomatis and Neisseria gonorrhoeae by use of nucleic acid amplification tests. J Clin Microbiol. 2009;47:1657–62.
[7] Ota KV, Tamari IE, Smieja M, Jamieson F, Jones KE, Towns L, et al. Detection of Neisseria gonorrhoeae and Chlamydia trachomatis in pharyngeal and rectal specimens using the BD Probetec ET system, the Gen-Probe Aptima Combo 2 assay and culture. Sex Transm Infect. 2009;85:182–6.
[8] Schachter J, Moncada J, Liska S, Shayevich C, Klausner JD. Nucleic acid amplification tests in the diagnosis of chlamydial and gonococcal infections of the oropharynx and rectum in men who have sex with men. Sex Transm Dis. 2008;35:637–42.
[9] Walsh A, Rourke FO, Crowley B. Molecular detection and confirmation of Neisseria gonorrhoeae in urogenital and extragenital specimens using the Abbott CT/NG RealTime assay and an in-house assay targeting the porA pseudogene. Eur J Clin Microbiol Infect Dis. 2011;30:561–7.
[10] Wind CM, de Vries HJ, Schim van der Loeff MF, Unemo M, van Dam AP. Successful combination of nucleic acid amplification test diagnostics and targeted deferred Neisseria gonorrhoeae culture. J Clin Microbiol. 2015;53:1884–90.
[11] Unemo M, Seifert HS, Hook EW, 3rd, Hawkes S, Ndowa F, Dillon JR. Gonorrhoea. Nat Rev Dis Primers. 2019;5:79.
