# Supplementary material for: Gonorrhoea: a systematic review of prevalence reporting globally
Source: BMC Infect Dis. 2021 Nov 11;21:1152. doi: 10.1186/s12879-021-06381-4 (PMC8582208; doi:10.1186/s12879-021-06381-4)
Supplement: Supplementary file 5 — Additional file 5. Reported gonorrhoea prevalence and/or test positivity in men-who-have-sex-with-men, by WHO region, country, and anatomic site. [file 12879_2021_6381_MOESM5_ESM.pdf]

Additional file 5. Reported gonorrhoea prevalence and/or test positivity in men-who-have-sex-with-men, by WHO region, country, and anatomic site

WHO region	Country	Reference	Years reported	Gonorrhoea prevalence										
				Urethral			Rectal			Pharyngeal			Any/All	
				No. tested	Reported prevalence and/or test positivity	Standardized estimate	No. tested	Reported prevalence and/or test positivity	Standardized estimate	No. tested	Reported prevalence and/or test positivity	Standardized estimate	Total No. tested	Reported prevalence and/or test positivity
**Africa**	Botswana	Tafuma et al [1]	2012	450	1.4%	1.5%	450	1.8%	0.0%	··	··	··	450	2.9%
	Kenya	Sanders et al [2]	2011	244	1.6%	1.8%	244	5.7%	4.0%	··	··	··	··	··
		Sanders et al [3]	2005–2011	449	2.0%	2.2%	449	0.4%	0.6%	··	··	··	··	··
	Nigeria	Keshinro et al [4]	2013–2016	862	3.8%	4.4%	862	23.3%	22.9%	··	··	··	862	24.2%
	Senegal	Wade et al [5]	2007	500	2.6%	2.9%	··	··	··	··	··	··	··	··
	South Africa	Rebe et al [6]	2012	200	3.5%	4.0%	200	8.5%	7.0%	200	7.5%	5.9%	200	16.0%
	Tanzania	Ross et al [7]	2012–2013	219	1.0%	1.1%	219	12.3%	11.1%	··	··	··	··	··
	Uganda	Kim et al [8]	2008–2009	288	1.4%	1.5%	286	1.8%	0.0%	··	··	··	··	··
**Americas (excluding high-income North America)**	Brazil	Cunha et al [9]	2010–2012	273	0.0%	0.0%	279	2.5%	0.5%	··	··	··	··	··
	El Salvador	Creswell et al [10]	2008	648	1.2%	1.3%	506	3.6%	1.7%	··	··	··	··	··
	Jamaica	Figueroa et al [11]	2007–2008	201	3.5%	4.0%	··	··	··	··	··	··	··	··
	Peru	Allan-Blitz et al [12][1]	2013–2014	··	··	··	387	8.8%	7.3%	387	6.5%	4.8%	··	··
		Castillo et al [13]	2009–2012	··	··	··	510	8.6%	7.1%	510	5.4%	3.7%	··	··
		Kojima et al [14]	2013–2014	··	··	··	312	8.4%	6.9%	312	5.8%	4.1%	312	14.2%
		Leon et al [15]	2008–2009	··	··	··	701	9.6%	8.2%	712	6.5%	4.8%	··	··
		Perez-Brumer et al [16]	2007	560	2.1%	2.3%	··	··	··	··	··	··	··	··
**Europe**	Germany	Dudareva-Vizule et al [17]	2009–2010	685	1.9%	2.1%	2050	4.6%	2.8%	2197	5.5%	3.8%	··	··
		Marcus et al [18]	2011–2012	··	··	··	642	3.6%	1.7%	882	5.4%	3.7%	··	··
	Ireland	Igoe et al [19]	2013	··	··	··	··	··	··	··	··	··	3286	11.1%
	Italy	Foschi et al [20]	2017	··	··	··	165	27.3%	27.2%	84	19.0%	18.3%	··	··
	Netherlands	Heiligenberg et al [21]	2008–2009	673	4.8%	3.8%	673	5.1%	3.3%	673	4.2%	2.4%	673	10.7%
		Van Liere et al [22]	2010–2012	2436	1.5%	1.7%	2436	3.7%	1.9%	2436	3.4%	1.5%	2436	6.3%
		Van Liere et al [23]	2011–2012	9534	1.9%	0.9%	9534	4.2%	2.4%	532	90.6%	95.4%	··	··

	UK	Haidari et al [24][2]	2010–2012	··	··	··	··	··	··	··	··	··	··	13.0%
		Soni et al [25]	2008	438	0.2%	0.2%	412	3.2%	5.0%	··	··	··	438	5.0%
**High-**	Canada	Gratrix et al [26][3]	2012	972	2.4%	2.7%	972	5.9%	4.2%	··	··	··	972	6.5%
**income**		Remis et al [27]	2010–2012	147	0.0%	0.0%	··	··	··	··	··	··	··	··
**North**	USA	Anschuetz et al [28]	2014–2015	2756	2.4%	2.7%	1763	5.2%	3.5%	2505	5.6%	3.8%	··	··
**America**		Beymer et al [29]	2011–2013	··	··	··	··	··	··	··	··	··	6367	14.5%
		Crosby et al [30]	··	··	··	··	··	··	··	··	··	··	346	7.7%
		Freeman et al [31]	2009	··	··	··	··	··	··	480	6.7%	5.0%	··	··
		Hassan et al [32]	2013–2015	··	··	··	395	21.3%	20.7%	··	··	··	··	··
		Mayer et al [33]	2004–2010	889	0.1%	0.0%	··	··	··	··	··	··	··	··
		Montano et al [34]	2013–2014[4]	183	6.6%	5.5%	183	27.9%	27.8%	183	18.0%	17.3%	183	11.1%
		Mustanski et al [35]	2013–2015	998	0.9%	0.9%	995	5.0%	3.3%	··	··	··	··	··
		Park et al [36]	2010	··	··	··	··	··	··	12 457	5.8%	4.1%	··	··
		Patton et al [37]	2010–2012	18 460	11.1%	11.1%[5]	11 092	10.2%	8.9%	14 484	7.9%	6.4%	··	··
		Pinsky et al [38]	2007–2010	··	··	··	··	··	··	200	3.5%	1.6%	··	··
		Sexton et al [39]	2009–2011	··	··	··	374	8.0%	6.5%	374	9.3%	7.9%	··	··
		Taylor et al [40]	2011–2013	··	··	··	1591	19.6%	19.0%	··	··	··	··	··
**South-East**	India	Aggarwal et al [41]	2013–2014	52[6]	5.8%	6.2%	··	··	··	··	··	··	··	··
**Asia**	Indonesia	Hananta et al [42]	2014	275	17.8%	16.7%	··	··	··	··	··	··	··	··.
		Morineau et al [43]	2007	749	1.9%	2.1%	738	18.6%	17.9%	··	··	··	··	··
	Thailand	Pattanasin et al [44]	2006–2010	1695	0.8%	0.9%	1552	5.9%	4.2%	1689	0.4%	0.0%	1695	6.4%
		Tongtoyai et al [45]	2006–2010	1743	1.8%	2.0%	1596	6.1%	4.5%	1740	0.5%	0.0%	··	··
**Western**	Australia	Cheung et al [46]	2007–2013	··	··	··	··	··	··	··	··	··	5256	27.8%
**Pacific**		Chow et al [47]	2015	··	··	··	··	··	··	823	10.1%	8.7%	··	··
		Chow et al [48]	2007–2013	25 766	2.3%	1.3%	22 746	2.9%	0.9%	25 233	1.7%	0.0%	··	··
		Nash et al [49]	2002–2012	37 553	1.7%	1.8%	··	··	··	··	··	··	··	··
		Ong et al [50]	2015–2016	5497	4.2%	4.7%	··	··	··	··	··	··	··	··
		Ryder et al [51]	2006–2008	5666	0.7%	0.0%	··	··	··	··	··	··	··	··
		Templeton et al [52]	2001–2004	··	··	··	··	··	··	1225	0.6%	0.0%	··	··

	Vodstrcil et al [53]	2002–2009	7611	4.2%	3.2%	5833	3.1%	3.1%[5]	6980	1.8%	0.0%	··	··
	Yang et al [54]	2015–2016	··	··	··	2392	10.5%	9.1%	2605	8.3%	6.7%	··	··
China[7]	Chen et al [55]	··	444	1.4%	1.4%[5]	··	··	··	··	··	··	··	··
	Fu et al [56]	2009	413	3.6%	4.1%	··	··	··	··	··	··	··	··
	Guo et al [57]	2007	··	··	··	··	··	··	··	··	··	139	15.8%
	Guo et al [58]	2009–2010	291	2.4%	2.7%	··	··	··	··	··	··	··	··
	Huan et al [59]	2010	··	··	··	328	3.7%	3.7%[5]	··	··	··	··	··
	Liu et al [60]	2009	1008	3.0%	3.0%[5]	··	··	··	··	··	··	··	··
	Yang et al [61]	2015–2017	463	5.2%	6.0%	463	6.1%	4.4%	463	3.9%	2.0%	463	12.5%
	Zhang et al [62]	2014–2015	296	1.4%	1.5%	296	6.8%	5.2%	296	8.1%	6.6%	296	13.2%
Korea (Rep. of)	Jung et al [63]	2008	106	0.0%	0.0%	··	··	··	··	··	··	··	··
Vietnam	Pham et al [64]	2009	381	1.8%	2.0%	··	··	··	··	··	··	··	··

If the standardized estimate was a negative number, the standardized prevalence was reported at 1 case divided by 100 times the sample size [65].

[1]85 (22%) study participants were transgender. The results for MSM and transgender were not reported separately.

[2]Proportion of MSM tested during routine testing at Department of Genitourinary Medicine. Denominator was not provided in this letter to the editor.

[3]Testing prevalence per screening visit is reported. Number of study participants not reported.

[4]The proportion of MSM with gonorrhoea over a 12-month period prior to starting pre-exposure prophylaxis is reported.

[5]Unable to apply laboratory standardization due to lack of information on laboratory testing.

[6]Given the rarity of data from India, this study was included despite a sample size of <100.

[7]Two additional studies were reported in a systematic review by Chow et al [66], for which we were unable to retrieve the original reports of the data. According to Chow et al, prevalence of gonorrhoea in MSM was reported to be 1.4% (1/70) in Yunnan Province in 2009 and 1.0% (1/105) in 2010 [67]; in 2008 in Nanning, prevalence of urethral infection in MSM was 0.9% (1/106) and of rectal infection 0% (0/130) [68].

··=not reported. MSM=men-who-have-sex-with-men. No=number. Rep.=Republic. UK=United Kingdom. USA= United States of America. WHO=World Health Organization.

References Additional file 5

[1] Tafuma TA, Merrigan MB, Okui LA, Lebelonyane R, Bolebantswe J, Mine M, et al. HIV/sexually transmitted infection prevalence and sexual behavior of men who have sex with men in 3 districts of Botswana: results from the 2012 biobehavioral survey. Sex Transm Dis. 2014;41:480–5.
[2] Sanders EJ, Wahome E, Okuku HS, Thiong'o AN, Smith AD, Duncan S, et al. Evaluation of WHO screening algorithm for the presumptive treatment of asymptomatic rectal gonorrhoea and chlamydia infections in at-risk MSM in Kenya. Sex Transm Infect. 2014;90:94–9.
[3] Sanders EJ, Okuku HS, Smith AD, Mwangome M, Wahome E, Fegan G, et al. High HIV-1 incidence, correlates of HIV-1 acquisition, and high viral loads following seroconversion among MSM. AIDS. 2013;27:437–46.
[4] Keshinro B, Crowell TA, Nowak RG, Adebajo S, Peel S, Gaydos CA, et al. High prevalence of HIV, chlamydia and gonorrhoea among men who have sex with men and transgender women attending trusted community centres in Abuja and Lagos, Nigeria. J Int AIDS Soc. 2016;19:21270.
[5] Wade AS, Larmarange J, Diop AK, Diop O, Gueye K, Marra A, et al. Reduction in risk-taking behaviors among MSM in Senegal between 2004 and 2007 and prevalence of HIV and other STIs. ELIHoS Project, ANRS 12139. AIDS Care. 2010;22:409–14.
[6] Rebe K, Lewis D, Myer L, de Swardt G, Struthers H, Kamkuemah M, et al. A cross sectional analysis of gonococcal and chlamydial infections among men-who-have-sex-with-men in Cape Town, South Africa. PLoS One. 2015;10:e0138315.
[7] Ross MW, Nyoni J, Ahaneku HO, Mbwambo J, McClelland RS, McCurdy SA. High HIV seroprevalence, rectal STIs and risky sexual behaviour in men who have sex with men in Dar es Salaam and Tanga, Tanzania. BMJ Open. 2014;4:e006175.
[8] Kim EJ, Hladik W, Barker J, Lubwama G, Sendagala S, Ssenkusu JM, et al. Sexually transmitted infections associated with alcohol use and HIV infection among men who have sex with men in Kampala, Uganda. Sex Transm Infect. 2016;92:240–5.
[9] Cunha CB, Friedman RK, de Boni RB, Gaydos C, Guimaraes MRC, Siqueira BH, et al. Chlamydia trachomatis, Neisseria gonorrhoeae and syphilis among men who have sex with men in Brazil. BMC Public Health. 2015;15:686.
[10] Creswell J, Guardado ME, Lee J, Nieto AI, Kim AA, Monterroso E, et al. HIV and STI control in El Salvador: results from an integrated behavioural survey among men who have sex with men. Sex Transm Infect. 2012;88:633–8.
[11] Figueroa JP, Weir SS, Jones-Cooper C, Byfield L, Hobbs MM, McKnight I, et al. High HIV prevalence among men who have sex with men in Jamaica is associated with social vulnerability and other sexually transmitted infections. West Indian Med J. 2013;62:286–91.
[12] Allan-Blitz LT, Leon SR, Bristow CC, Konda KA, Vargas SK, Flores JA, et al. High prevalence of extra-genital chlamydial or gonococcal infections among men who have sex with men and transgender women in Lima, Peru. Int J STD AIDS. 2017;28:138–44.
[13] Castillo R, Konda KA, Leon SR, Silva-Santisteban A, Salazar X, Klausner JD, et al. HIV and sexually transmitted infection incidence and associated risk factors among high-risk MSM and male-to-female transgender women in Lima, Peru. J Acquir Immune Defic Syndr. 2015;69:567–75.
[14] Kojima N, Park H, Konda KA, Joseph Davey DL, Bristow CC, Brown B, et al. The PICASSO Cohort: baseline characteristics of a cohort of men who have sex with men and male-to-female transgender women at high risk for syphilis infection in Lima, Peru. BMC Infect Dis. 2017;17:255.
[15] Leon SR, Segura ER, Konda KA, Flores JA, Silva-Santisteban A, Galea JT, et al. High prevalence of Chlamydia trachomatis and Neisseria gonorrhoeae infections in anal and pharyngeal sites among a community-based sample of men who have sex with men and transgender women in Lima, Peru. BMJ Open. 2016;6:e008245.
[16] Perez-Brumer AG, Konda KA, Salvatierra HJ, Segura ER, Hall ER, Montano SM, et al. Prevalence of HIV, STIs, and risk behaviors in a cross-sectional community- and clinic-based sample of men who have sex with men (MSM) in Lima, Peru. PLoS One. 2013;8:e59072.
[17] Dudareva-Vizule S, Haar K, Sailer A, Wisplinghoff H, Wisplinghoff F, Marcus U, et al. Prevalence of pharyngeal and rectal Chlamydia trachomatis and Neisseria gonorrhoeae infections among men who have sex with men in Germany. Sex Transm Infect. 2014;90:46–51.
[18] Marcus U, Ort J, Grenz M, Eckstein K, Wirtz K, Wille A. Risk factors for HIV and STI diagnosis in a community-based HIV/STI testing and counselling site for men having sex with men (MSM) in a large German city in 2011-2012. BMC Infect Dis. 2015;15:14.

[19] Igoe D, Kelleher M, Cooney F, Clarke S, Quinlan M, Lyons F, et al. There has been a true rise in Neisseria gonorrhoeae but not in Chlamydia trachomatis in men who have sex with men in Dublin, Ireland. Sex Transm Infect. 2014;90:523.
[20] Foschi C, Gaspari V, Sgubbi P, Salvo M, D'Antuono A, Marangoni A. Sexually transmitted rectal infections in a cohort of 'men having sex with men'. J Med Microbiol. 2018;67:1050–7.
[21] Heiligenberg M, Wermeling PR, van Rooijen MS, Urbanus AT, Speksnijder AGCL, Heijman T, et al. Recreational drug use during sex and sexually transmitted infections among clients of a city sexually transmitted infections clinic in Amsterdam, the Netherlands. Sex Transm Dis. 2012;39:518–27.
[22] van Liere GAFS, Hoebe CJPA, Dukers-Muijrers NHTM. Evaluation of the anatomical site distribution of chlamydia and gonorrhoea in men who have sex with men and in high-risk women by routine testing: cross-sectional study revealing missed opportunities for treatment strategies. Sex Transm Infect. 2014;90:58–60.
[23] van Liere GAFS, van Rooijen MS, Hoebe CJPA, Heijman T, de Vries HJ, Dukers-Muijrers NHTM. Prevalence of and factors associated with rectal-only chlamydia and gonorrhoea in women and in men who have sex with men. PLoS One. 2015;10:e0140297.
[24] Haidari G, Perry ME, White JA. Are we seeing a true rise in Neisseria gonorrhoeae and Chlamydia trachomatis in men who have sex with men in the U.K.? Sex Transm Infect. 2014;90:308.
[25] Soni S, Alexander S, Verlander N, Saunders P, Richardson D, Fisher M, et al. The prevalence of urethral and rectal Mycoplasma genitalium and its associations in men who have sex with men attending a genitourinary medicine clinic. Sex Transm Infect. 2010;86:21–4.
[26] Gratrix J, Singh AE, Bergman J, Egan C, McGinnis J, Drews SJ, et al. Prevalence and characteristics of rectal chlamydia and gonorrhea cases among men who have sex with men after the introduction of nucleic acid amplification test screening at 2 Canadian sexually transmitted infection clinics. Sex Transm Dis. 2014;41:589–91.
[27] Remis RS, Liu J, Loutfy MR, Tharao W, Rebbapragada A, Huibner S, et al. Prevalence and correlates of HIV infection and sexually transmitted infections in female sex workers (FSWs) in Shanghai, China. PLoS One. 2016;11:e0158090.
[28] Anschuetz GL, Paulukonis E, Powers R, Asbel LE. Extragenital screening in men who have sex with men diagnoses more chlamydia and gonorrhea cases than urine testing alone. Sex Transm Dis. 2016;43:299–301.
[29] Beymer MR, Weiss RE, Bolan RK, Rudy ET, Bourque LB, Rodriguez JP, et al. Sex on demand: geosocial networking phone apps and risk of sexually transmitted infections among a cross-sectional sample of men who have sex with men in Los Angeles County. Sex Transm Infect. 2014;90:567–72.
[30] Crosby RA, Graham CA, Mena L, Yarber WL, Sanders SA, Milhausen RR, et al. Circumcision status is not associated with condom use and prevalence of sexually transmitted infections among young black MSM. AIDS Behav. 2016;20:2538–42.
[31] Freeman AH, Bernstein KT, Kohn RP, Philip S, Rauch LM, Klausner JD. Evaluation of self-collected versus clinician-collected swabs for the detection of Chlamydia trachomatis and Neisseria gonorrhoeae pharyngeal infection among men who have sex with men. Sex Transm Dis. 2011;38:1036–9.
[32] Hassan A, Blumenthal JS, Dube MP, Ellorin E, Corado K, Moore DJ, et al. Effect of rectal douching/enema on rectal gonorrhoea and chlamydia among a cohort of men who have sex with men on HIV pre-exposure prophylaxis. Sex Transm Infect. 2018;94:508–14.
[33] Mayer KH, Ducharme R, Zaller ND, Chan PA, Case P, Abbott D, et al. Unprotected sex, underestimated risk, undiagnosed HIV and sexually transmitted diseases among men who have sex with men accessing testing services in a New England bathhouse. J Acquir Immune Defic Syndr. 2012;59:194–8.
[34] Montano MA, Dombrowski JC, Dasgupta S, Golden MR, Duerr A, Manhart LE, et al. Changes in sexual behavior and STI diagnoses among MSM initiating PrEP in a clinic setting. AIDS Behav. 2019;23:548–55.
[35] Mustanski B, Feinstein BA, Madkins K, Sullivan P, Swann G. Prevalence and risk factors for rectal and urethral sexually transmitted infections from self-collected samples among young men who have sex with men participating in the Keep It Up! 2.0 randomized controlled trial. Sex Transm Dis. 2017;44:483–8.
[36] Park J, Marcus JL, Pandori M, Snell A, Philip SS, Bernstein KT. Sentinel surveillance for pharyngeal chlamydia and gonorrhea among men who have sex with men--San Francisco, 2010. Sex Transm Dis. 2012;39:482–4.
[37] Patton ME, Kidd S, Llata E, Stenger M, Braxton J, Asbel L, et al. Extragenital gonorrhea and chlamydia testing and infection among men who have sex with men--STD Surveillance Network, United States, 2010-2012. Clin Infect Dis. 2014;58:1564–70.
[38] Pinsky L, Chiarilli DB, Klausner JD, Kull RM, O'Keefe R, Heffer C, et al. Rates of asymptomatic nonurethral gonorrhea and chlamydia in a population of university men who have sex with men. J Am Coll Health. 2012;60:481–4.

[39] Sexton ME, Baker JJ, Nakagawa K, Li Y, Perkins R, Slack RS, et al. How reliable is self-testing for gonorrhea and chlamydia among men who have sex with men? J Fam Pract. 2013;62:70–8.
[40] Taylor MM, Newman DR, Gonzalez J, Skinner J, Khurana R, Mickey T. HIV status and viral loads among men testing positive for rectal gonorrhoea and chlamydia, Maricopa County, Arizona, USA, 2011-2013. HIV Med. 2015;16:249–54.
[41] Aggarwal P, Bhattar S, Sahani SK, Bhalla P, Garg VK. Sexually transmitted infections and HIV in self reporting men who have sex with men: A two-year study from India. J Infect Public Health. 2016;9:564–70.
[42] Hananta IP, van Dam AP, Bruisten SM, Schim van der Loeff MF, Soebono H, de Vries HJ. Gonorrhea in Indonesia: high prevalence of asymptomatic urogenital gonorrhea but no circulating extended spectrum cephalosporins-resistant Neisseria gonorrhoeae strains in Jakarta, Yogyakarta, and Denpasar, Indonesia. Sex Transm Dis. 2016;43:608–16.
[43] Morineau G, Nugrahini N, Riono P, Nurhayati, Girault P, Mustikawati DE, et al. Sexual risk taking, STI and HIV prevalence among men who have sex with men in six Indonesian cities. AIDS Behav. 2011;15:1033–44.
[44] Pattanasin S, Dunne EF, Wasinrapee P, Tongtoyai J, Chonwattana W, Sriporn A, et al. Screening for Chlamydia trachomatis and Neisseria gonorrhoeae infection among asymptomatic men who have sex with men in Bangkok, Thailand. Int J STD AIDS. 2018;29:577–87.
[45] Tongtoyai J, Todd CS, Chonwattana W, Pattanasin S, Chaikummao S, Varangrat A, et al. Prevalence and correlates of Chlamydia trachomatis and Neisseria gonorrhoeae by anatomic site among urban Thai men who have sex with men. Sex Transm Dis. 2015;42:440–9.
[46] Cheung KT, Fairley CK, Read TRH, Denham I, Fehler G, Bradshaw CS, et al. HIV incidence and predictors of incident HIV among men who have sex with men attending a sexual health clinic in Melbourne, Australia. PLoS One. 2016;11:e0156160.
[47] Chow EPF, Walker S, Read TRH, Chen MY, Bradshaw CS, Fairley CK. Self-reported use of mouthwash and pharyngeal gonorrhoea detection by nucleic acid amplification test. Sex Transm Dis. 2017;44:593–5.
[48] Chow EPF, Tomnay J, Fehler G, Whiley D, Read TR, Denham I, et al. Substantial increases in chlamydia and gonorrhea positivity unexplained by changes in individual-level sexual behaviors among men who have sex with men in an Australian sexual health service from 2007 to 2013. Sex Transm Dis. 2015;42:81–7.
[49] Nash JL, Hocking JS, Read TR, Chen MY, Bradshaw CS, Forcey DS, et al. Contribution of sexual practices (other than anal sex) to bacterial sexually transmitted infection transmission in men who have sex with men: a cross-sectional analysis using electronic health records. Sex Transm Infect. 2014;90:55–7.
[50] Ong JJ, Fethers K, Howden BP, Fairley CK, Chow EPF, Williamson DA, et al. Asymptomatic and symptomatic urethral gonorrhoea in men who have sex with men attending a sexual health service. Clin Microbiol Infect. 2017;23:555–9.
[51] Ryder N, Lockart IG, Bourne C. Is screening asymptomatic men who have sex with men for urethral gonorrhoea worthwhile? Sex Health. 2010;7:90–1.
[52] Templeton DJ, Jin F, McNally LP, Imrie JC, Prestage GP, Donovan B, et al. Prevalence, incidence and risk factors for pharyngeal gonorrhoea in a community-based HIV-negative cohort of homosexual men in Sydney, Australia. Sex Transm Infect. 2010;86:90–6.
[53] Vodstrcil LA, Fairley CK, Fehler G, Leslie D, Walker J, Bradshaw CS, et al. Trends in chlamydia and gonorrhea positivity among heterosexual men and men who have sex with men attending a large urban sexual health service in Australia, 2002-2009. BMC Infect Dis. 2011;11:158.
[54] Yang TZT, Chen MY, Read TRH, Needleman R, Bradshaw CS, Fortune R, et al. Sampling technique and detection rates of oropharyngeal and anorectal gonorrhoea using nucleic acid amplification tests in men who have sex with men. Sex Transm Infect. 2018;94:287–92.
[55] Chen X, Fu G, Xu X, Hu H, Zuo H, Liu X, et al. [Study on gonococcal and chlamydial infections among men who have sex with men in Nanjin]. Acta Universitatis Medicinalis Anhui. 2011:569–72.
[56] Fu GF, Jiang N, Hu HY, Mahapatra T, Yin YP, Mahapatra S, et al. The epidemic of HIV, syphilis, chlamydia and gonorrhea and the correlates of sexual transmitted infections among men who have sex with men in Jiangsu, China, 2009. PLoS One. 2015;10:e0118863.
[57] Guo W, Wu ZY, Song AJ, Poundstone K. Impact of HIV/sexually transmitted infection testing on risky sexual behaviors among men who have sex with men in Langfang, China. Chin Med J (Engl). 2013;126:1257–63.
[58] Guo Y, Wang D, Zhou J, Chen S, Wang J, Zhen S, et al. [Effects of education level of men who have sex with men on their high risk sexual behaviors and the infection of HIV and syphilis]. Zhonghua Yu Fang Yi Xue Za Zhi. 2014;48:307–11.

[59] Huan XP, Yin YP, Fu GF, Jiang N, Zhang QQ, Zhang XN, et al. [Analysis on sexually transmitted diseases and the related risk factors among men who have sex with men in Jiangsu province]. Zhonghua Yu Fang Yi Xue Za Zhi. 2011;45:975–8.
[60] Liu YJ, Jiang SL, Hu Y, Song L, Yu M, Li SM. [Characteristics of sexual behaviors and infection status of AIDS and other sexually transmitted diseases among men who have sex with men in 2009 in Beijing]. Zhonghua Yu Fang Yi Xue Za Zhi. 2011;45:971–4.
[61] Yang LG, Zhang XH, Zhao PZ, Chen ZY, Ke WJ, Ren XQ, et al. Gonorrhea and chlamydia prevalence in different anatomical sites among men who have sex with men: a cross-sectional study in Guangzhou, China. BMC Infect Dis. 2018;18:675.
[62] Zhang X, Jia M, Chen M, Luo H, Chen H, Luo W, et al. Prevalence and the associated risk factors of HIV, STIs and HBV among men who have sex with men in Kunming, China. Int J STD AIDS. 2017;28:1115–23.
[63] Jung M, Lee J, Kwon DS, Park BJ. Comparison of sexual risky factors of men who have sex with men and sex-buying men as groups vulnerable to sexually transmitted diseases. J Prev Med Public Health. 2012;45:156–63.
[64] Pham QD, Nguyen TV, Hoang CQ, Cao V, Khuu NV, Phan HT, et al. Prevalence of HIV/STIs and associated factors among men who have sex with men in An Giang, Vietnam. Sex Transm Dis. 2012;39:799–806.
[65] Spectrum. Glastonbury: Avenir Health 2019. https://www.avenirhealth.org/software-spectrum.php. Accessed 30 November 2020.
[66] Chow EP, Tucker JD, Wong FY, Nehl EJ, Wang Y, Zhuang X, et al. Disparities and risks of sexually transmissible infections among men who have sex with men in China: a meta-analysis and data synthesis. PLoS One. 2014;9:e89959.
[67] Gao LM, Chen L, Ma Y, Lu JB, Li LX, Zhang YL. [HIV/STD infection and KABP status among men who have sex with men in Yuxi City, Yunnan Province, 2009-2010]. Soft Science of Health. 2010;24:547–9.
[68] Chen SH, Zhu JQ, Yang NH. [Investigation on HIV and STI infections among men who have sex with men in Nanning City during 2006–2008.]. Occupation and Health. 2010;26:56–8.
